# Differentiation of *Salmonella* strains from the SARA, SARB and SARC reference collections by using three genes PCR-RFLP and the 2100 Agilent Bioanalyzer

**DOI:** 10.3389/fmicb.2014.00417

**Published:** 2014-08-11

**Authors:** Ángel A. Soler-García, Antonio J. De Jesús, Kishana Taylor, Eric W. Brown

**Affiliations:** Molecular Methods and Subtyping Branch, Division of Microbiology, Center for Food Safety and Applied Nutrition, US Food and Drug AdministrationCollege Park, MD, USA

**Keywords:** *Salmonella enterica*, Bioanalyzer, PCR-RFLP, restriction type, reference collection

## Abstract

Rapid molecular typing methods are important tools in surveillance and outbreak investigations of human *Salmonella* infections. Here we described the development of a three-genes PCR-RFLP typing method for the differentiation of *Salmonella* species, subspecies and serovars using the Agilent 2100 Bioanalyzer. The *fliC*, *gnd*, and *mutS* genes were PCR-amplified in 160 *Salmonella* strains representing the two *Salmonella* species, six subspecies, and 41 different serovars of *S. enterica* subspecies *enterica*. PCR products were individually cut with two different restriction enzymes and the resulting 930 restriction patterns were collected using the Agilent 2100 Bioanalyzer followed by cluster analysis. Both species of *Salmonella* were differentiated by conventional PCR. All of *S. bongori* tested were *gnd* PCR negative due to a mismatch at the 3′-end in one the PCR primers. *Salmonella* subspecies were differentiated into third-teen homogeneous groups representing each of the six subspecies by cluster analysis of restriction patterns generated from the *mutS* gene cut with *AciI*. *S. enterica* subspecies *enterica* serovars were further differentiated by the combination of the three target genes and five out the six sets of restriction patterns with a discriminatory power of 0.9725 by cluster analysis. The combined RFLP results of five sets of restriction patterns allowed us to assign each of the 160 strains to one of 128 restriction types. During inoculation studies we were able to identify *S*. Saintpaul and Typhimurium from 24 h pre-enrichment samples using the described method. The use of *fliC*, *gnd*, and *mutS* PCR-RFLP with the Agilent 2100 Bioanalyzer can provide an accessible and automated alternative method for differentiation of *Salmonella* pathogens.

## Introduction

Contaminated food consumed in the United States causes an estimated 48 million illnesses, 128,000 hospitalizations, and 3,000 deaths annually (Scallan et al., 2011a,b). *Salmonella* alone causes approximately 1 million foodborne infections (Scallan et al., 2011b), 19,336 hospitalizations, 378 deaths annually (CDC, 2011a) with a cost of $365 million in direct medical expenditure (CDC, 2011b). Human salmonellosis is one of the most frequently occurring food-borne diseases worldwide (Wattiau et al., 2011). Foods prepared with contaminated raw eggs, egg products, insufficiently heated poultry meat and pork have been identified as the primary sources of human *Salmonella* infections (Buchholz et al., 2005). Although non-typhoid *Salmonella* strains commonly cause self-limiting gastroenteritis, severe infections, including bacteremia and meningitis, have also been reported (Sirinavin et al., 1999). A combination of sanitary measures and surveillance programs monitoring the entire food chain (animal feed, living animals, slaughterhouses, retail sector, and restaurants) in a timely manner are essential for the detection and prevention of human *Salmonella* infections (Bertrand et al., 2010). Success depends upon having rapid and sensitive methods for the detection and characterization of *Salmonella*. Work to develop and improve these methods may lessen the disease burden caused by this pathogen.

*Salmonella* is divided into two different species, *S. enterica* and *S. bongori*. *S. enterica* itself consists of six subspecies, *enterica* (I), *salamae* (II), *arizonae* (IIIa), *diarizonae* (IIIb), *houtenae* (IV), and *indica* (VI) forming a diverse group 2,557 serovars (Tindall et al., 2005; Grimont and Weill, 2007). Of the six subspecies, only members of subspecies *enterica* are associated with disease in warm-blooded animals and only a small fraction of these frequently cause disease in humans and domestic animals. The classical methods for identifying and typing *S. enterica* isolates consist of phenotypic methods that include biochemical profiling, serotyping and phage typing (Andrews et al., 2007; Grimont and Weill, 2007). The gold standard for *Salmonella* serotyping is based on the scheme developed by Kuffman, White, and Le Minor (Grimont and Weill, 2007). Serotyping deciphers the antigenic makeup of the organisms by identifying the somatic (O) and flagellar (H) antigens through reactions with specific antisera and is useful for international surveillance programs (Herikstad et al., 2002). However, traditional serotyping is unable to adequately fingerprint strains, therefore molecular typing has become primary tool for understanding the evolution of *Salmonella* and trace clones with special traits such as antibiotic resistance (Herikstad et al., 2002; Foley et al., 2007, 2009).

The current gold standard for molecular typing is Pulse-Field Gel Electrophoresis (PFGE), which can provide discrimination between similar serotypes and is the basis for PulseNet surveillance (Schwartz and Cantor, 1984). However, PFGE is laborious, time-consuming, and expensive. A subtyping method should be rapid, robust, portable, and sensitive. It should be able to reliably differentiate epidemiologically unrelated strains from each other and group all isolates associated with the same source without disrupting the present classification of *Salmonella* into subspecies and serovars. Such a subtyping system would also need to work within budget constraints of laboratories. For these reasons, we explored ways to improve existing techniques using an accessible platform that can be widely-distributed.

Polymerase Chain Reaction-Restriction Fragment Length Polymorphism (PCR-RFLP) is a variation of RFLP in which a specific PCR product is amplified followed by restriction digestion with restriction endonucleases to generate a specific restriction banding pattern (Owen and Leeton, 1999). For adequate discrimination the amplified region or gene needs to have a variable region flanked by conserved regions to allow PCR amplification and generation of different restriction patterns after cutting restriction enzymes. Restriction patterns are analyzed using a conventional agarose gel followed by gel documentation to analyze the resolved banding patterns. PCR-RFLP has been used previously for the serotyping of *Salmonella* (Kilger and Grimont, 1993; Shah and Romick, 1997; Dauga et al., 1998; Kwon et al., 2000; Matsui et al., 2001; Hong et al., 2003; Kisiela et al., 2005; Albarnaz et al., 2007; Gallegos-Robles et al., 2008; Hu et al., 2009). Several targets such as ribosomal (Albarnaz et al., 2007), *groEL* (Hu et al., 2009), *fimA* (Kisiela et al., 2005) and *recA* (Matsui et al., 2001) genes have been used for the differentiation of *Salmonella* subspecies and serovars. For this purpose *fliC* has been the most targeted gene (Kilger and Grimont, 1993; Shah and Romick, 1997; Dauga et al., 1998; Kwon et al., 2000; Hong et al., 2003; Gallegos-Robles et al., 2008). By relying on one region of the genome or specific gene, the technique is limited in its discriminatory power, and by the possibility of ambiguous bands on a conventional agarose gel. Previous studies have demonstrated improved accuracy and reproducibility of RFLP using the 2100 Agilent Bioanalyzer for the sizing of the DNA fragments (Panaro et al., 2000; Nachamkin et al., 2001; Lu et al., 2002; Hathaway et al., 2007). In this study, we test the utility of the 2100 Agilent Bioanalyzer for differentiating *Salmonella* species, subspecies and serovars using PCR-RFLP of the of *fliC*, *gnd*, and *mutS* genes.

## Materials and methods

### *Salmonella* strains and target genes for molecular serotyping

**Figure 1:**
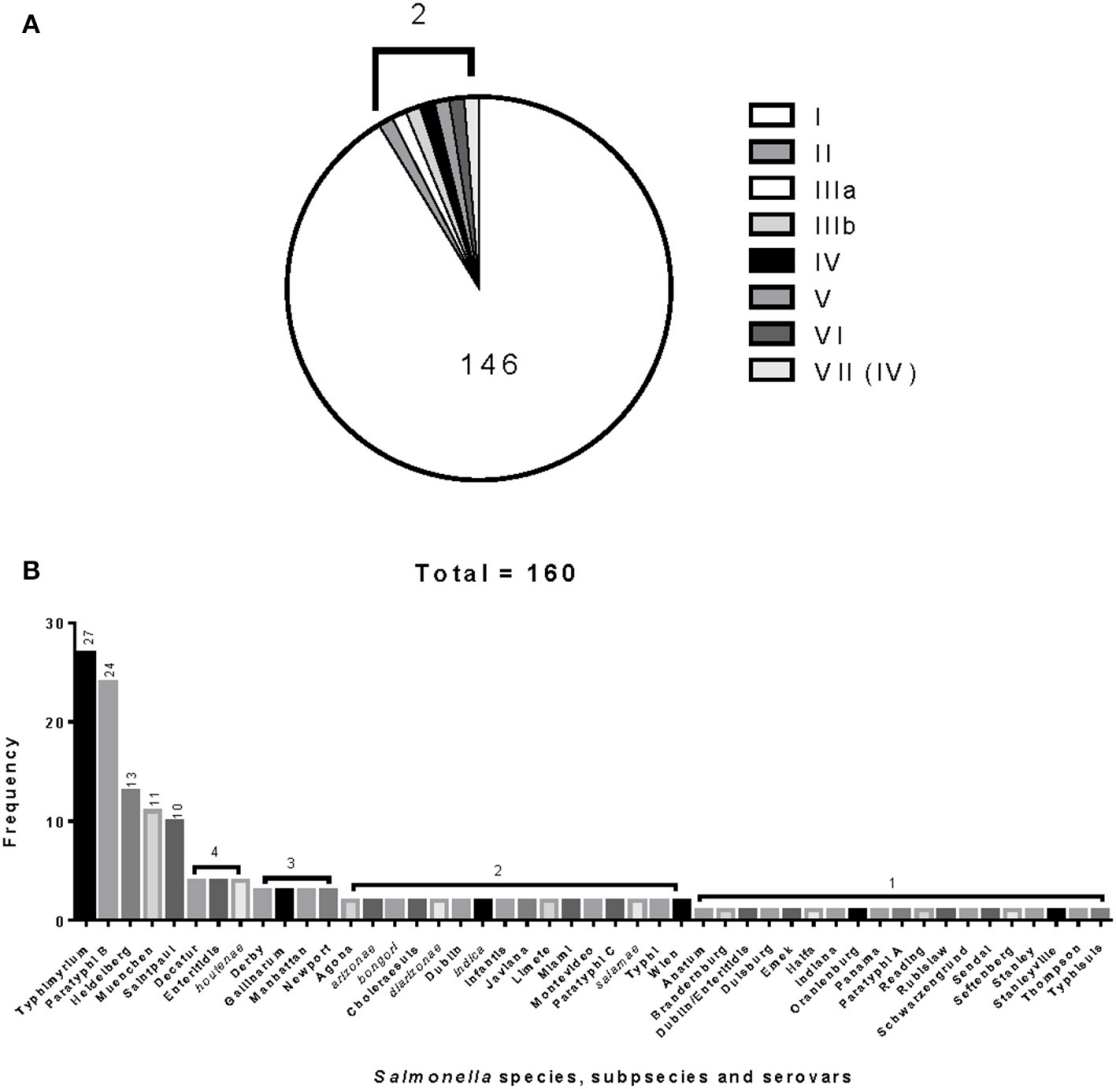
**Distribution and frequencies *Salmonella* species, subspecies and serovars**. The 160 *Salmonella* strains tested belong to three different *Salmonella* Reference (SAR) collections A, B and C. **(A)** Describes the distribution and frequency of *Salmonella* species and subspecies. **(B)** Shows the distribution and frequencies of the *Salmonella* species, subspecies, and serovars.

The 160 reference strains included in this study represent 41 different serovars of *S. enterica* subsp. *enterica* (subsp. I). The six *Salmonella* subspecies belong to the *Salmonella* Reference Collections SAR A (72), B (72), and C (16) (Beltran et al., 1991; Boyd et al., 1993, 1996). Recently corrections have been made to certain serovars in the SAR A and B collections (Achtman et al., 2013). Figures 1A,B show the distribution of *Salmonella* species, subspecies and serovars. The *Salmonella fliC*, *gnd* and *mutS* genes were selected as candidate targets for the development of the PCR-RFLP. The *fliC* gene encodes for the phase 1 flagellar antigen and it is present in all *Salmonellae* (Mcquiston et al., 2004). For the phase 1 antigen, 52 antigenic factors and 61 serotypes (single factors or combinations of factors) have been distinguished (Li et al., 1994). The *gnd* gene codes for 6-phosphogluconate, an enzyme of the pentose-phosphate pathway, and is located between the *rfb* locus and the highly variable *cld* gene (Nelson and Selander, 1994; Thampapillai et al., 1994). The *mutS* gene, a key component of the methyl-directed mismatch repair system, acts as barrier to horizontal gene transfer by blocking recombination of diverged DNA (Brown et al., 2002, 2003).

### Preparation of DNA and PCR amplification of *fliC*, *gnd* and *mutS* genes

Isolates were grown in Tryptic Soy agar (TSA) plates (Difco, BD, Sparks, MD). A single colony was grown in a shaking incubator overnight at 37°C in Brain Heart Infusion broth (BHIB) (Difco). One ml of the broth culture was transferred to a 1.5 ml microcentrifuge tube and centrifuged at 12,000 rpm for 3 min. Total genomic DNA was isolated using the Promega Wizard Genomic DNA Purification kit (Promega Corporation, Madison, WI). The primers used for the amplification of the selected genes are described in Table S1. DNA amplification by PCR was performed in a reaction volume of 50 μl consisting of 25 μl of Qiagen Hot StarTaq Plus master mix (Qiagen, Valencia, CA), 20 μM primer mix, 10 ng of total genomic DNA and volume was completed with molecular biology grade water. Initial denaturation was carried out for 5 min at 95°C. Thirty cycles of amplification were performed in a DNA Engine Tetrad2 Peltier Thermal Cycler (Bio-Rad, Hercules, CA). Each cycle consisted of three steps: denaturation for 30 sec at 94°C, annealing for 30 s at 60°C, and extension for 1 min at 72°C. An additional step of extension for 7 min at 72°C was performed at the end of the amplification to complete extension of the primers. Amplification products were detected by resolving 1 μl of the PCR product using the Agilent DNA 7500 kit and the 2100 Agilent Bioanalyzer (Agilent Technologies, Inc., Santa Clara, CA).

### Restriction digestion and validation of PCR-RFLP database for sequenced *Salmonella* genomes

We used the *In silico* (http://insilico.ehu.es) database to virtually test PCR primers and select possible restriction enzymes to test experimentally during RFLP (Bikandi et al., 2004; Roberts et al., 2007). The *Salmonella* database consisted of 27 genomes representing 15 species (*S. bongori*), subspecies (*S. enterica* subsp. *arizonae*) and *S. enterica* serovars: Agona, Choleraesuis, Dublin, Enteritidis, Gallinarum-Pullorum (2), Heidelberg (2), Newport, Paratyphi A (2), Paratyphi B, Paratyphi C, Schwarzengrund, Typhi (3) and Typhimurium (8). Enzymes showing the most number of different restriction patterns among the 27 *Salmonella* genomes were selected for pilot experiments.

The PCR products were cut using the following restriction enzymes: *fliC* was cut with *HhaI* and *Sau3AI*; *gnd* with *AciI* and *AluI*; and *mutS* with *AciI* and *HaeII*. Restriction enzymes from New England Biolabs (Ipswich, MA) and Fermentas (Glen Burnie, MD) were used during the development of the molecular typing method. Single digestions were done by mixing 5 μl of the selected PCR product and 2.5U of NEB endonucleases or 1 Fast digest unit of the Fermentas endonucleases in final volume of 10 μl. NEB endonuclease mixtures were incubated for 1 h at 37°C. Fast digest mixtures were incubated for 10 min at 37°C. After incubation, DNA digestion was terminated by heat inactivation at 65°C for 20 or 10 min depending on the enzyme used or by the addition of 20 mM EDTA. In selected experiments, restriction digestions were cleaned using the MinElute Reaction Cleanup kit (Qiagen). Restriction digestions were repeated two to three times to test reproducibility of the restriction patterns. Restriction patterns were analyzed using the Agilent DNA 1000 kit and the 2100 Agilent Bioanalyzer (Agilent Technologies, Inc.).

### RFLP cluster analysis, sequencing of *fliC*, *gnd*, *mutS*, and MLST housekeeping genes

Data files containing RFLP patterns from the 2100 Agilent Bioanalyzer were exported as data set tables in CSV format. These were then imported into BioNumerics version 6.6 (Applied Maths, Inc., Austin, TX). The relationships between restriction patterns were calculated by cluster analysis for each and/or combination of restriction patterns using Ward and DICE coefficient with optimization of 1% and tolerance of 0.25%. Ward and Dice were used as recommended by the Guidelines for the validation and application of typing methods for use in bacterial epidemiology (Van Belkum et al., 2007). All the nucleotide sequencing was performed in both directions through MCLAB (San Francisco, CA) and assembled into single complete sequences using the CLC Main Workbench software version 6.8.2 (Aarhus, DK). The *fliC*, *gnd*, and *mutS* genes were sequenced using the primers in Table S1 in all *Salmonella* collections. The *mutS* in the SAR B; *mutS* and *gnd* in the SAR C collections were obtained from GenBank (NCBI) in FASTA format (Brown et al., 2002, 2003). Sequences for the seven MLST housekeeping genes, *aroC*, *dnaN*, *hemD*, *hisD*, *purE*, *sucA*, and *thrA* for SARA and B collections were obtained from the NCBI database (Bell et al., 2011) and the MLST Databases at the ERI, University College Cork, respectively. MLST was performed on the 16 *Salmonella* strains composing the SAR C collection (Boyd et al., 1996; Maiden et al., 1998; Enright and Spratt, 1999). All primers sequences of the seven MLST genes, for amplification and sequencing are described in Table S1. These primers contain M13/pUCR forward (5′-CCCAGTCACGACGTTGTAAAACG-3′) and reverse (5′-AGCGGATAACAATTTCACACAGGAA-3′) universal sequencing priming sites.

PCR cycling conditions were as follows. Initial denaturation was carried out for 5 min at 95°C. Thirty-five cycles of amplification were performed in a DNA Engine Tetrad2 Peltier Thermal Cycler (Bio-Rad). Each cycle consisted of three steps: denaturation for 1 min at 94°C, annealing for 1 min at 55°C, and extension for 1 min at 72°C. An additional step of extension for 5 min at 72°C was performed at the end of the amplification to allow complete extension of the primers. Amplification products were detected by resolving 1 μl of the PCR product using the Agilent DNA 1000 kit and the 2100 Agilent Bioanalyzer (Agilent Technologies, Inc.).

The *fliC*, *gnd*, and *mutS* genes were aligned using BioEdit version 7.1.11, and trimmed with GeneStudio version 2.2.0.0. The seven housekeeping genes sequences were aligned with allele templates from the MLST Database, and then aligned and trimmed as described above. Then, the sequences were queried to the MLST Database website for allele number assignment. Concatenated analyses of *fliC*, *gnd*, and *mutS*; and the seven housekeeping genes were conducted using MEGA software version 5.0.5, using neighbor-joining method with Tamura-Nei distance and 1000 bootstrapping replicates (Felsenstein, 1985; Saitou and Nei, 1987; Tamura et al., 2011).

### Preparation of *Salmonella* inocula, artificial inoculation and analysis of food commodities

*Salmonella enterica* serovars Newport, Saintpaul, and Typhimurium were selected for artificial contamination of alfalfa sprouts, jalapeno peppers and tomatoes, respectively (CDC, 2006, 2008, 2010). These serovars have been previously implicated in outbreaks related to these food commodities. *Salmonella* inocula for artificial contamination of produce were prepared as described before (Zhang et al., 2011). Alfalfa sprouts, jalapeno peppers and tomatoes were obtained from local supermarkets. These food commodities were processed as described before (Zhang et al., 2011). Briefly, for each *Salmonella* serovar and their corresponding produce, two 25 g portions of food were placed aseptically into a sterile Seward stomacher bag (Seward, United Kingdom). The two portions were designated as A, for no inoculation and B, for high-level inoculation (10^5^ CFU/ml). The jalapeno peppers and tomatoes were chopped aseptically in a blender into sizes similar to what is present in regular, chunky salsa and then weighed before being placed into preenrichment bags. One ml of the selected *Salmonella* serovar at the indicated concentration was added to the 25 g produce portion. For the no inoculated control one ml of the MDR buffer was used. Bags were massaged gently by hand for 1 min and kept at 4°C for 2 h. For enrichment, 225 ml of universal enrichment broth (Difco) were added to the bags. Bags were then shaken vigorously by hand for 30 s, and incubated (without shaking) at 35 ± 1°C for 24 ± 1 h.

One ml aliquots were taken from each bag (A and B) for DNA extraction, serial dilutions further microbiological testing. Four different DNA extraction methods were tested. First, a 1 ml sample was heated at 100°C for 12 min and then centrifuged for 2 min at 16,000× *g* (Eppendorf, New York). One ml samples were centrifuged and the pellets resuspended in 100 μl of sterile distilled water and boiled, or DNA was extracted using either the Epicenter Quick DNA extraction (Madison, WI) and the Promega Wizard Genomic DNA Purification following the instructions of their manufacturers. Samples were stored at −20°C. Microbiological analysis of 24 h per-enrichment samples was as previously described (Andrews et al., 2007; Zhang et al., 2011). Identification and confirmation of *Salmonella* were done using Biolog GEN III plates (Biolog, Inc.; Hayward, CA). *Salmonella* serotyping was done following the standard protocol for molecular determination of serotype in *Salmonella* based on the Bioplex technology (Fitzgerald et al., 2007; Mcquiston et al., 2011).

All primers and probes used in this study were purchased from IDT (Coralville, IA) and are given in Table S1. Real-time PCR was done as described before (Deer et al., 2010). Briefly, qPCRs were done using the QuantiFast Multiplex PCR using all the DNA templates following the recommended protocol (Qiagen). Each 25 μl reaction contained 1× Master Mix (HotStarTaq Plus DNA Polymerase, QuantiFast Multiplex PCR Buffer, and dNTP mix), 400 nmol/l IAC primers, 200 nmol/l IAC probe and 1 μl DNA IAC template (1 · 1 pg/μl). For the multiplex reactions, *invA* primers, invA_176F and invA_291R; and probe invA_Tx_208 were added at 200 and 150 nmol/l, respectively. The qPCR conditions were as follows: 95°C for 5 min (for polymerase activation) and 40 cycles of 95°C for 45 s and 60°C for 45 s with fluorescence acquisition for both, Cy5 and Texas Red, following each 60°C step. All qPCR assays were run in CFX96 Real-Time System (Bio-Rad). The term *C*q is equivalent to the original *CT* (threshold cycle) terminology according to the Minimum Information for Publication of Quantitative Real- Time PCR Experiments (MIQE) guidelines (Bustin et al., 2009, 2010). Conventional PCR for *fliC*, *gnd*, and *mutS* was done as described in the Materials and Methods Section.

## Results

### PCR-amplification of *fliC*, *gnd* and *mutS Salmonella* genes and validation of *in silico* PCR amplification tool

**Figure 2:**
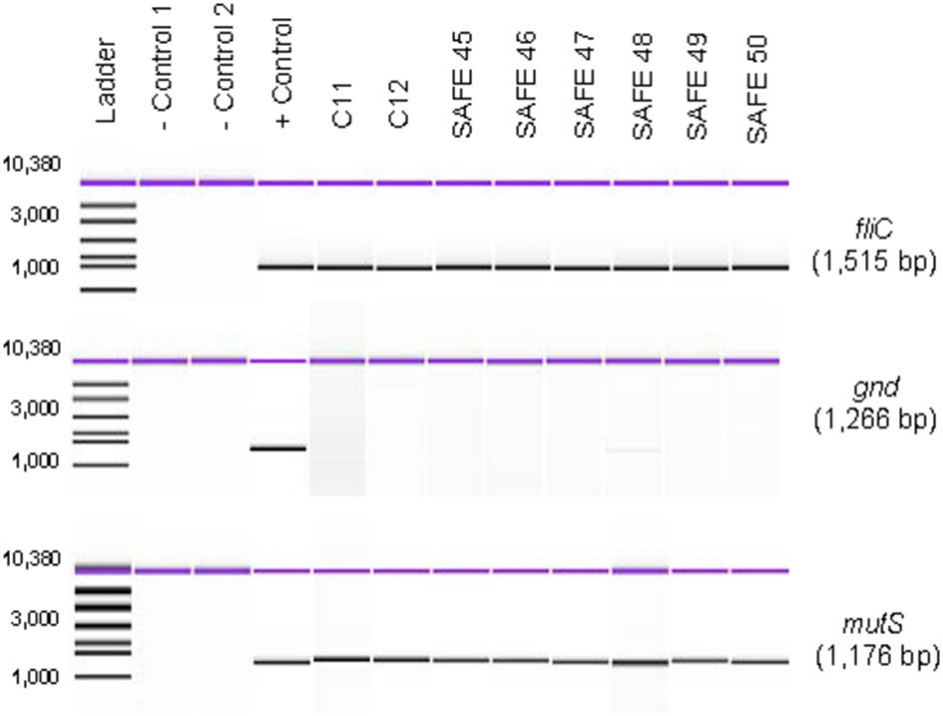
**Absence of *gnd* gene in *S. bongori* strains**. The *gnd* gene was PCR-amplified as described in the Materials and Methods from eight different strains of *S. bongori*. PCR products were resolved using the Agilent 7500 DNA kit and the Agilent 2100 Bioanalyzer. Negative Control 1 corresponds to PCR reagents mix + water as a template; and Negative Control 2 corresponds to DNA from a known *gnd* negative bacterial strain using our PCR conditions.

Virtual PCR was done using the designed *fliC*, *gnd*, and *mutS* specific gene primers against the *Salmonella* database (Table S1). The virtual analysis showed all (100%) of the *Salmonella* strains were PCR positive for the *fliC* gene (Table 1). However, the virtual PCR simulation predicted negative results for three out of 27 (11%) database strains for the *gnd* and *mutS* genes: *S. bongori* str. NCTC 12419, *S. enterica* subsp. *arizonae* 62:z4,z23:- and *S. enterica* subsp. enterica serovar Newport str. SL254 (Table 1). Experimental PCR confirmed *fliC* amplification in all the tested strains. Contrary to the PCR database predictions, *S. bongori* (2) and S. *enterica* subsp. *arizonae* (2) were PCR positive for the *mutS* gene. As previously reported, one out of the three *S. enterica* subsp. *enterica* serovar Newport (SAR B37) was PCR negative for *mutS* (Brown et al., 2003). In agreement with the *In silico* database, 100% of the *S. bongori* (2) were PCR negative for the *gnd* gene experimentally (Table 1 and Figure 2).

**Table 1:** **PCR-amplification of *fliC*, *gnd*, and *mutS* genes in *Salmonella* species, subspecies and serovars**.

**Gene**	**PCR amplification**					
	**Virtual (*In silico*)**			**Experimental**		
	**Positive (%)**	**Negative (%)**	**Isolates**	**Positive (%)**	**Negative (%)**	**Isolates**
*fliC*	27/27	0/27		160/160	0/160	
	(100)	(0)		(100)	(0)	
*gnd*	24/27	3/27	*S. bongori*	158/160	2/160	C11-12
	(89)	(11)	*S. arizonae*	(98.8)	(1.2)	*S. bongori*
			*S*. Newport			
*mutS*	24/27	3/27	*S. bongori*	159/160	1/160	B37
	(89)	(11)	*S. arizonae*	(99.3)	(0.7)	*S*. Newport
			*S*. Newport			

### Differentiation of *S. enterica* from *S. bongori*

To confirm our experimental results, we tested six more strains of *S. bongori* belonging to the Systems and Assays for Food Examination (SAFE) Reference Collection (Mcquiston et al., 2008): 94-0708 (V 48:i:-), 95-0123 (V 40:z35:-), 96-0233 (V 44:z39:-), CNM-256 (V 60:z41:-), CNM262 (V 66:z41:-), 95-0321(V 48:z35:-), and repeated strains SAR C11 and 12 for PCR amplification of the *gnd* gene. One hundred % of the of *S. bongori* were PCR negative for the *gnd* gene under our experimental PCR conditions (Figure 2). These data validate the predicted results from the *In silico* website with respect to *S. bongori*. A possible cause for the lack of amplification of the *gnd* gene in *S. bongori* strains can be primer-template mismatches. Mismatches located in the 3′-end region of a primer have significantly larger effects on priming efficiency than mismatches located at the 5′-end (Beard et al., 2004; Johnson and Beese, 2004; Stadhouders et al., 2010). We identified mistmatches in the 3′-end of the *gnd* F-1 primer (Table S1) by aligning 16 *gnd Salmonella* gene sequences from the SAR C Reference Collection (Boyd et al., 1996) (**Figure S1**). This mismatch resulted in the differentiation of *S. bongori* strains from the other *Salmonella* specie and subspecies. To confirm the specificity of these results, with conducted an exclusivity PCR test (defined here as the lack of the signal or negative reaction on closely related non-*Salmonella* strains) against a panel of 20 different gram-positive and gram-negative bacterial strains (Table S2). In this test, 100% of the exclusivity strains tested were PCR negative for the *fliC* and *gnd* genes. Five out 20 (25%) showed lower levels of *mutS* PCR product as compared to a *Salmonella* positive control. A PCR profile with positive *fliC* and *mutS* genes in combination with a negative PCR amplification of the *gnd* gene suggests the presence of *S. bongori*, while S. *enterica* will exhibit a positive PCR profile for the three genes. These data suggest that the two species of *Salmonella* can be differentiated by PCR using the described three PCR amplification profile.

### Selection of restriction enzymes for experimental RFLP and validation of *Salmonella* RFLP database for sequenced genomes

To select the restriction enzymes to be used for the RFLP experimentally, we conducted virtual RFLP of *fliC*, *gnd*, and *mutS* genes for 27 *Salmonella* sequenced strains in the *In silico* database. Analysis of the banding patterns showed several enzymes that produced four or more different restriction patterns per specific gene tested. Pairs of such restriction enzymes were chosen specifically for each of the three genes to generate experimental restriction patterns: *fliC* gene, *Hha*I and *Sau3AI*; *gnd*, *AciI* and *AluI*; and *mutS*, *AciI* and *HaeII*.

Given the fact that we had four out the 27 *Salmonella* strains with complete genomes in the database, we decided to validate the predicted RFLP patterns. We used the data obtained from the following sequenced available genomes: *S. enterica* subsp. *arizonae* 62:z4,z23:- (SAR C5), serovar Paratyphi A str. ATCC 9150 (SAR B42), Paratyphi C str. RKS4595 (SAR B49) and Typhimurium str. LT2 (SAR A2). The predicted restriction patterns were compared to the experimental data generated using the 2100 Agilent Bioanalyzer (Table S3). The degree of agreement between the predicted number of fragments and the total size, and our experimental RFLP for the four *Salmonella* strains was evaluated. All (100%) of the simulated restriction patterns were different as compared to the experimental ones. The differences in total size of the predicted and the experimental fragments varied from 0.6 to 10.4%.

### Processing and reproducibility of DNA restriction patterns

**Figure 3:**
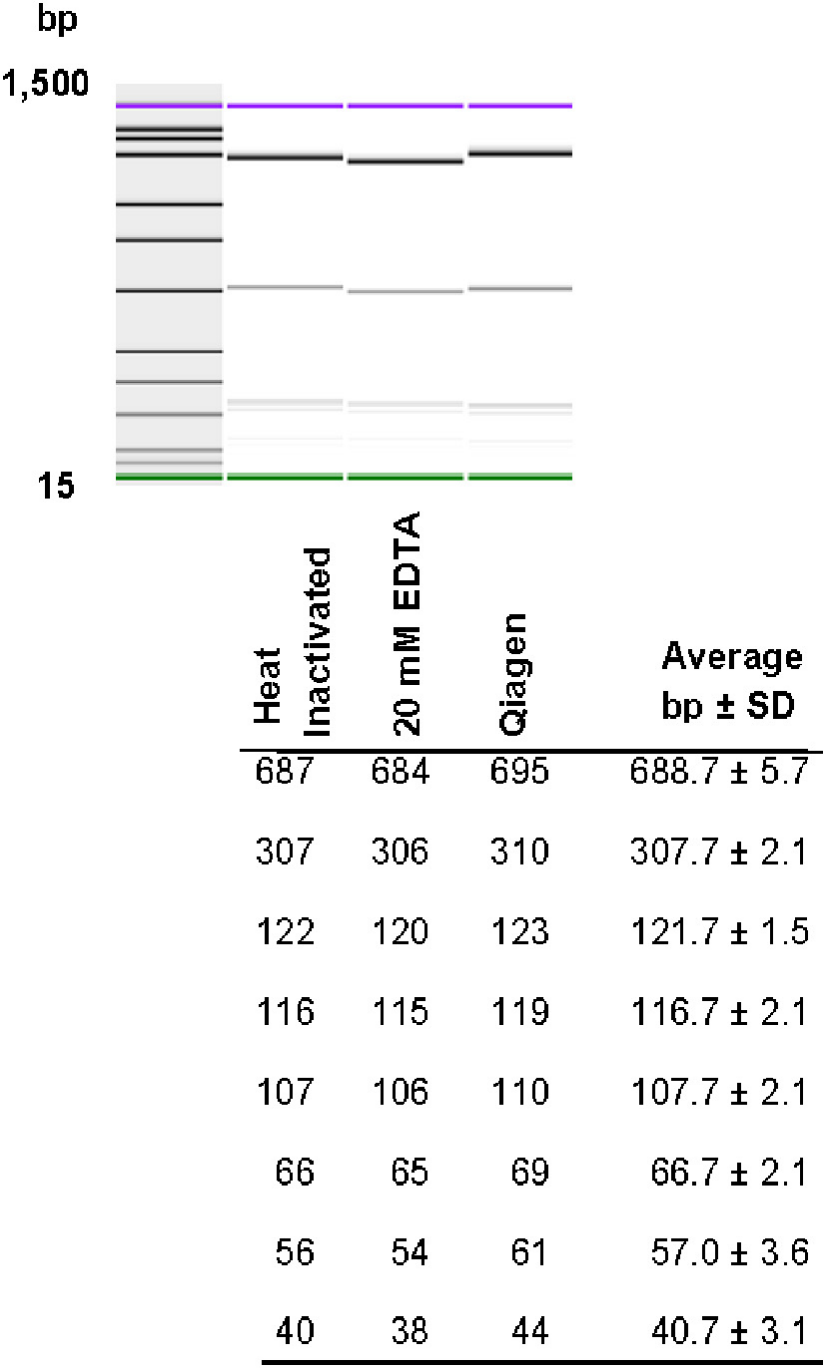
**Effect of different restriction enzyme inactivation methods in the number and sizes of restriction fragments**. The *fliC* gene of *S*. Typhimurium was PCR-amplified and cut with *HhaI* restriction enzyme as described in the Materials and Methods. Restriction enzyme activity was stop by heat inactivation, addition of 20 mM EDTA or the reaction was cleaned with a commercially available kit.

Restriction patterns were resolved using the Agilent DNA 1000 kit (Agilent Technologies). This kit reports a sizing accuracy of ±10%, depending upon the fragment size range, and a sizing resolution that varies from ±5 bp, ±5% and ±10% in the fragments ranging from 25–100, 100–500, and 500–1000 bp, respectively (Agilent Technologies). When resolving restricted DNA using the Bioanalyzer, adding EDTA and/or using heat inactivation of the restriction enzymes is recommended to avoid possible degradation of the internal DNA marker (Agilent Technologies). We tested the effect of adding 20 mM EDTA, heat inactivation, and the use of a commercially-available method for cleaning restriction digestion reactions on the resolution of restriction fragments and reproducibility of restriction patterns in the 2100 Agilent Bioanalyzer. No significant differences in the number of restriction fragments or the sizes obtained among treatments were observed (Figure 3). Although minor differences were detected among fragment sizes between 2 and 5 bp, no degradation of the 1500 and 15 bp internal markers were observed (Figure 3). Given these results, we chose the use of 20 mM EDTA for the inactivation of the restriction enzymes. Although heat inactivation is a cheaper alternative, this step adds 10–20 min to the procedure depending on the restriction enzyme in use.

### Differentiation of *Salmonellae* by RFLP cluster analysis

**Figure 4:**
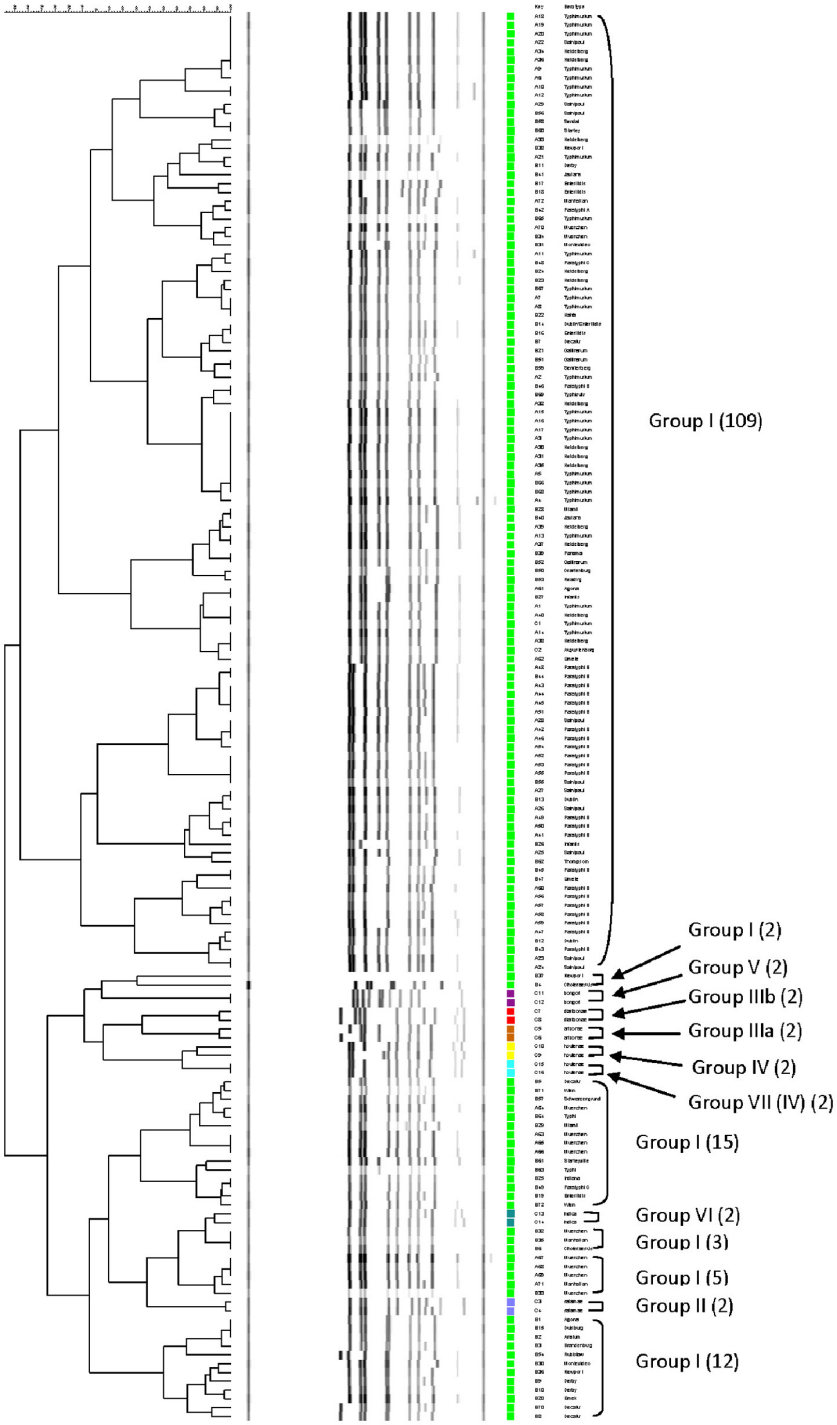
**Differentiation of *Salmonella* species and subspecies by PCR-RFLP and cluster analysis**. The *mutS* gene was PCR-amplified on the 160 *Salmonella* strains. The PCR product was cut with *AciI*. Restriction patterns were analyzed as described in the Materials and Methods. The relationship among restriction patterns was analyzed by cluster analysis using BioNumerics. The *mutS*-*AciI* banding pattern is shown. Homogeneous clusters consisting of *Salmonella* subspecies are indicated.

The relationship among restriction patterns was analyzed by cluster analysis. A total of possible 63 individual and multiple combinations were analyzed. Dendrograms were drawn using BioNumerics (Applied Maths). *S. enterica* is comprised of six subspecies: *enterica* (I), *salamae* (II), *arizonae* (IIIa), *diarizonae* (IIIb), *houtenae* (IV), and *indica* (VI). For simplicity, *S. bongori* is still commonly referred to as subsp. V (Tindall et al., 2005). Some derivatives of *S. enterica* subsp. *houtenae* (IV) have been reported and identified as subgroup VII (Boyd et al., 1996). Based on biotype these are very similar to subsp. IV but can be distinguished by multilocus enzyme electrophoresis (Boyd et al., 1996). To establish whether PCR-RFLP has the potential to differentiate among *Salmonella* subspecies we conducted cluster analysis as described in the Material and Methods Section. Based on the distribution of subspecies and the number of members in each subspecies group (Figure 1A), we expected that the best clustering would consist of seven to eight clusters depending on whether the derivatives of subsp. IV could be separated in two distinct clusters. These theoretical subspecies clustering show a discriminatory power (DP) equal to 0.167 (Hunter and Gaston, 1988; Hunter, 1990). The current RFLP cluster analysis showed that restriction patterns obtained cutting the *mutS* gene with the restriction enzyme *AciI* was indeed sufficient to differentiate the different subspecies of *Salmonella* (Figure 4). *S. enterica* subsp. II, IIIa, IIIb, IV, V, and VI were grouped in single homogeneous clusters (Figure 4). *S. enterica* subsp. I was grouped into six homogeneous clusters consisting of 109, 2, 15, 3, 5, and 12 members, respectively. This clustering corresponds to a DP of 0.5219. This suggests that *Salmonella* subspecies can be differentiated by *mutS*-*AciI* RFLP cluster analysis.

There are a total of 2,579 serovars in the genus *Salmonella* distributed between the two species and six subspecies, the bulk of which (1531 serovars) are in *S. enterica* subsp. *enterica* (Grimont and Weill, 2007). The current study represents 41 serovars of S. *enterica* subsp. *enterica* (Figure 1B). To establish whether PCR-RFLP has the potential to differentiate among *Salmonella* species, subspecies and serovars, we conducted cluster analysis as described in the Material and Methods Section. Based on the distribution of S. *enterica* subsp. *enterica* serovars and the five subspecies we expect that the best clustering for our subset should consist of the following: 28 homogeneous clusters containing at least two representatives of a selected serovar or subspecies, and 19 individual serovars (due to their representation with 1 member), for a total of 47 different types among the 160 strains. This clustering distribution corresponds to a DP of 0.9331 (Hunter and Gaston, 1988; Hunter, 1990).

**Figure 5:**
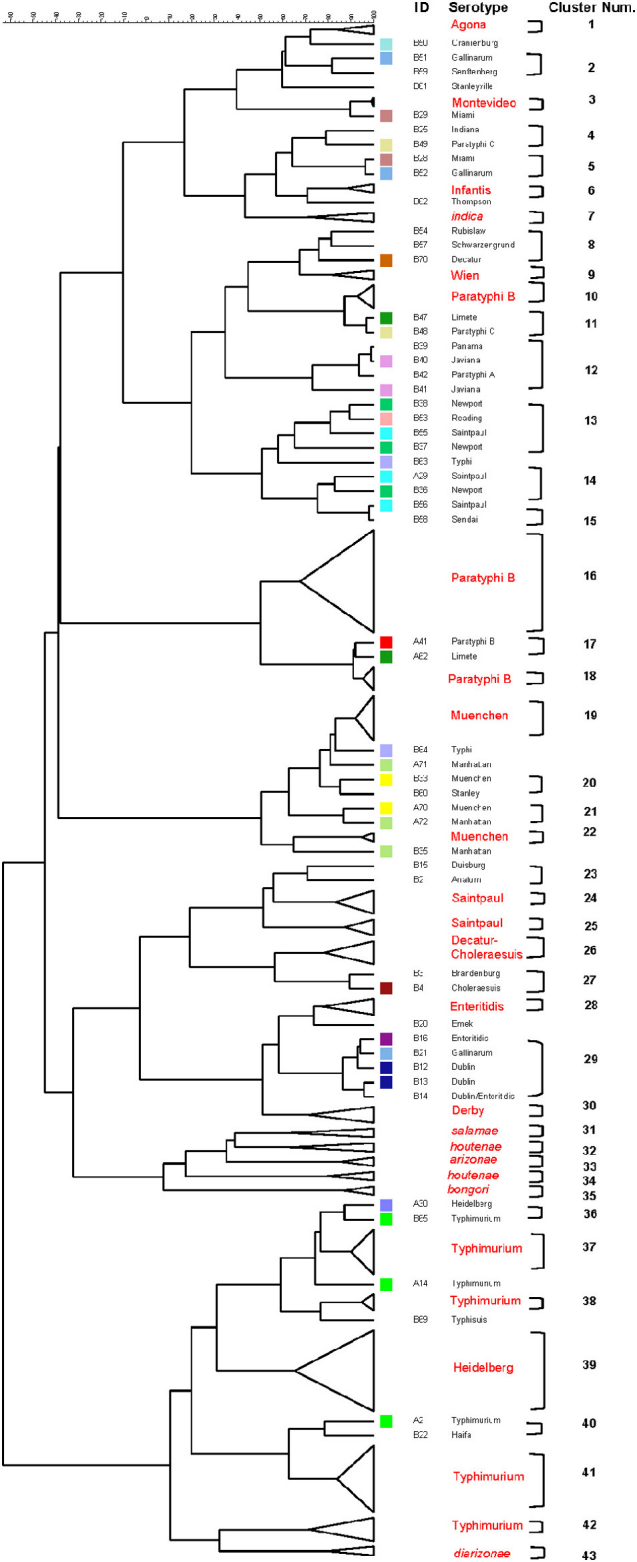
**Differentiation of *Salmonella* species, subspecies and serovars by PCR-RFLP and cluster analysis**. The *fliC*, *gnd*, *mutS* genes were PCR-amplified on the 160 *Salmonella* strains. The PCR products were cut with specific restriction enzymes and the relationship among restriction patterns were analyzed as described in the Material and Methods. Homogeneous clusters consisting of one *Salmonella* species, subspecies, and serovars are identified in red.

The six restrictions patterns obtained by the digestion of the *fliC*, *gnd*, and *mutS* genes were analyzed in BioNumerics. We obtained best differential clustering using the combination of the *fliC* gene cut with *HhaI* and *Sau3AI*; *gnd* gene cut with *AciI* and *AluI*; the *mutS* gene cut with *HaeII*. Forty-three different clusters and eleven single serovars were identified for a total of 54 different types (Figure 5). This cluster distribution corresponds to a DP of 0.9725. The 43 clusters and the relationship among strains on each cluster are described in Table 2. Twenty-six out of 43 (60.5%) clusters consisted of different homogeneous serovar groups. Nineteen out of the 28 (68%) serovars and/or subspecies represented by more than one strain were grouped into homogeneous clusters (Table 2). In twelve out of these 19 (63.2%) homogeneous clusters representing serovars and subspecies containing more than one strain, 100% of the representing strains were grouped together. Seventeen out of 43 (39.5%) clusters were defined as Type I clusters consisting of *S. enterica* subsp. *enterica* strains. Five (29.4%), five (29.4%) and eight (47%) of the 17 Type I clusters did not share, shared one or two elements in their antigenic formula, respectively. Four out of five (80%) clusters sharing one element shared either H1 or H2 antigen. Five out of eight (62.5%) clusters sharing two elements shared the O and the H2 antigen. Thirty-seven % (3/8) shared both H1 and H2 antigens.

**Table 2:** **Three-genes PCR-RFLP clusters**.

**Cluster ID**	**Num./Total**	**% Clustered**	**Serovar/Subspecie**	**Group**	**Shared element of antigenic formula**			**O group**
					**O**	**H1**	**H2**	
1	2/2	100	Agona	I	1,4,[5],12	f,g,s	[1,2]	O:4
2	2/146	1.4		I			–	
3	2/2	100	Montevideo	I	{6,7,14}{54}	g,m,[p],s	[1,2,7]	O:7
4	2/146	1.4		I				
5	2/146	1.4		I	1,9,12			O:9
6	2/2	100	Infantis	I	6,7,14	r	1,5	O:7
7	2/2	100	*Indica*	VI			e,n,x	
8	3/146	2.0		I				
9	2/2	100	Wien	I	1,4,12,27	b	l,w	O:4
10	4/24	17	Paratyphi B	I	1,4,[5],12	b	1,2	O:4
11	2/146	1.4		I			1,5	
12	4/146	2.7		I	1,#,12		1,5	
13	4/146	2.7		I		e,h		
14	2/146	1.4		I		e,h	1,2	
15	2/146	1.4		I				
16	15/24	63	Paratyphi B	I	1,4,[5],12	b	1,2	O:4
17	2/146	1.4		I		b		
18	4/24	17	Paratyphi B	I	1,4,[5],12	b	1,2	O:4
19	7/11	64	Muenchen	I	6,8	d	1,2	O:8
20	2/146	1.4		I		d	1,2	
21	2/146	1.4		I	6,8	d		O:8
22	2/11	18	Muenchen	I	6,8	d	1,2	O:8
23	2/146	1.4		I				
24	4/10	40	Saintpaul	I	1,4,[5],12	e,h	1,2	O:4
25	3/10	30	Saintpaul	I	1,4,[5],12	e,h	1,2	O:4
26	4/7	57	Decatur-Choleraesuis	I	6,7	c	1,5	O:7
27	2/146	1.4		I				
28	3/4	75	Enteritidis	I	1,9,12	g,m	–	O:9
29	5/146	3.4		I	1,9,12		–	O:9
30	3/3	100	Derby	I	1,4,[5],12	f,g	[1,2]	O:4
31	2/2	100	*salamae*	II				
32	2/2	100	*houtenae*	IV			–	
33	2/2	100	*arizonae*	IIIa	62		–	O:62
34	2/2	100	*houtenae*	VII			–	O:42
35	2/2	100	*bongori*	V			–	
36	2/146	1.4		I	1,4,[5],12		1,2	O:4
37	7/27	25.9	Typhimurium	I	1,4,[5],12	i	1,2	O:4
38	3/27	11.1	Typhimurium	I	1,4,[5],12	i	1,2	O:4
39	12/13	92	Heidelberg	I	1,4,[5],12	r	1,2	O:4
40	2/146	1.4		I	1,4,[5],12		1,2	O:4
41	10/27	37	Typhimurium	I	1,4,[5],12	i	1,2	O:4
42	4/27	14.8	Typhimurium	I	1,4,[5],12	i	1,2	O:4
43	2/2	100	*diarizonae*	IIIb				

*^#^Extra element not shared by members of this cluster*.

*^_^underlined is to indicate the presence of an O factor due to phage conversion*.

*^{}^O factors within curly brackets indicate that factors in curly brackets cannot coexist with others factors in curly brackets*.

*^[]^O or H factor that may be present or absent without relation to phage conversion*.

### Detection of *Salmonella* in artificially inoculated produce

To test the applicability of the PCR-RFLP in the detection of *S. enterica* subsp. *enterica* in contaminated produce after 24 h of pre-enrichment, we artificially inoculated food commodities with *Salmonella* serovars known to have been responsible for past outbreaks associated with the those food commodities: alfalfa sprouts with *S*. Newport, jalapeno peppers with *S*. Saintpaul, and tomatoes with *S*. Typhimurium (CDC, 2006, 2008, 2010). Food commodities were inoculated as described before the Materials and Methods Section. Four 1 ml aliquots were collected after 24 h pre-enrichment and different DNA extraction methods were tested to assess the effect of these different extraction methods on the amplification of the *fliC*, *gnd*, and *mutS* genes. We used a *Salmonella*-specific qPCR as a comparator (Deer et al., 2010). Conventional PCRs of *fliC*, *gnd* and *mutS* genes were affected by the DNA extraction and the food commodity (data not shown). *Salmonella* spp. was detected by qPCR in all DNA extraction methods in all food commodities tested. However, a positive amplification of the three RFLP genes by conventional PCR was obtained using the commercially available DNA extraction kit in jalapeño peppers and tomatoes. In tomatoes, pelleting bacteria from pre-enrichment followed by resuspension in water and boiling was also a good source for DNA template for conventional PCR. Although amplification of the three RFLP genes was observed using DNA extracted with the commercially available kit from alfalfa sprouts pre-enrichment, the yield of PCR product was too low for further manipulation (data not shown). Restriction patterns obtained from pre-enrichment samples exhibited identical patterns when compared to pure culture controls.

We next compared our PCR-RFLP method with the BAM standard method (Andrews et al., 2007). After artificial inoculation *Salmonella* strains were detected by the BAM standard method, confirmed by biochemical fingerprint (Biolog, Inc.) and serotyped using the *Salmonella* standard molecular serotyping method (Table 3). Using our previously established cluster analysis, PCR-RFLP identified and serotyped *S*. Saintpaul and *S*. Typhimurium after 24 h pre-enrichment (Table 3). However, low amplification yield for *S*. Newport precluded further manipulation of three RFLP genes. Following the BAM Method, all three *Salmonella* strains used for the artificially inoculation were isolated, identified and serotyped from all food commodities.

**Table 3:** **Summary of artificially inoculated food commodities**.

**Produce**	**Serotype (Id)**	**Inoculum (cfu)**	**Detection Method**					
			**qPCR**		**BAM**	**Biolog**	**Bioplex**	**PCR-RFLP**
			**IC (Cq)**	***Salmonella* spp. (Cq)**	**Culture**			
Alfalfa	Newport (SAR B37)	−Control	20.55 ± 0.30	−(0.0)		ND	ND	ND
		+Control	20.58 ± 0.13	+(22.33 ± 0.00)	NT	S. *enterica*	S. Newport	S. Newport
		10^5^	21.82 ± 2.97	+(23.09 ± 0.16)	+	*S. enterica*	S. Newport	ND
Jalapeno pepper	Saintpaul (SAR A22)	−Control	20.94 ± 0.19	−(0.0)		ND	ND	ND
		+Control	21.30 ± 0.03	+(18.41 ± 0.28)	NT	S. *enterica*	S. Saintpaul	S. Saintpaul
		10^5^	21.08 ± 0.03	+(16.42 ± 0.19)	+	*S. enterica*	S. Saintpaul	S. Saintpaul
Tomato	Typhimurium (SAR A1)	−Control	21.04 ± 0.18	−(0.0)		ND	ND	ND
		+Control	21.18 ± 0.04	+(18.30 ± 0.80)	NT	S. *enterica*	S. Typhimurium	S. Typhimurium
		10^5^	21.07 ± 0.35	+(13.23 ± 0.77)	+	*S. enterica*	S. Typhimurium	S. Typhimurium

*ND, no detected; NT, no tested; − and +, negative and positive detection for *Salmonella* spp., respectively*.

### Differentiation of *Salmonellae* by concatenated sequence analyses of mlst housekeeping, *fliC*, *gnd*, and *mutS* genes

**Figure 6:**
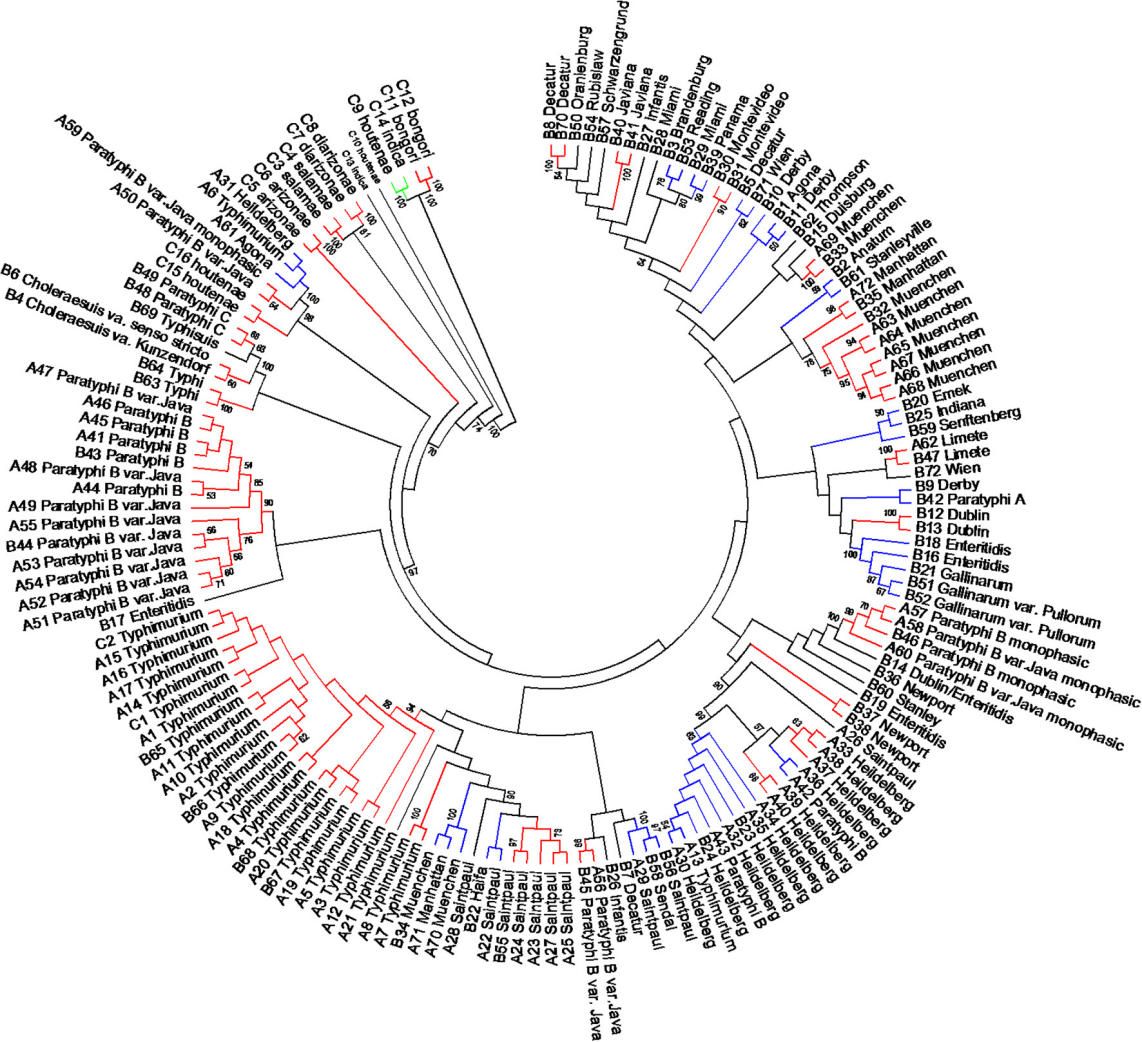
**Concatenated seven housekeeping genes sequences evolutionary relationships of taxa**. The evolutionary history was inferred using the Neighbor-Joining method (Saitou and Nei, 1987). The bootstrap consensus tree inferred from 1000 replicates is taken to represent the evolutionary history of the taxa analyzed (Felsenstein, 1985). Branches corresponding to partitions reproduced in less than 50% bootstrap replicates are collapsed. The evolutionary distances were computed using the Maximum Composite Likelihood method (Tamura et al., 2004) and are in the units of the number of base substitutions per site. The analysis involved 160 nucleotide sequences. All positions containing gaps and missing data were eliminated. There were a total of 3010 positions in the final dataset. Evolutionary analyses were conducted in MEGA5 (Tamura et al., 2011). Specific, Type I and mix clusters are represented in red, blue, and green, respectively.

The length of the seven concatenated housekeeping genes was 3,138 bp. The number of variable nucleotides was 59.4% among the 160 *Salmonella* species, subspecies, and serovars. Evolutionary analyses were conducted in MEGA5 (Tamura et al., 2011). The evolutionary history was inferred using the Neighbor-Joining method (Saitou and Nei, 1987). The evolutionary distances were computed using the Maximum Composite Likelihood method (Tamura et al., 2004) and are in the units of the number of base substitutions per site (Figure 6). Forty-one different clusters and 21 single serovars were identified for a total of 62 different types (Figure 6). This cluster distribution corresponds to a DP of 0.9652. The 41 clusters and the relationship among strains on each cluster are described in Table 4. Twenty-six out of 41 (63.4%) clusters consisted of different homogeneous serovar groups. Twenty out of the 28 (71%) serovars and/or subspecies represented by more than one strain were grouped into homogeneous clusters (Table 4). In eleven out of these 20 (52.4%) homogeneous clusters representing serovars and subspecies containing more than one strain, 100% of the representing strains were grouped together. Fourteen out of 41 (34.1%) clusters were defined as Type I clusters consisting of *S. enterica* subsp. *enterica* strains. Five (35.7%), one (7.1%) and eight (57.1%) of the 14 Type I clusters did not share, shared one or two elements in their antigenic formula, respectively. Seven out of eight (87.5%) clusters sharing two elements shared O and the H2 antigen. The remaining cluster sharing two elements of the antigenic formula was composed of strains sharing the O and the H1 (1/8; 12.5%). Only one cluster out of 41 (2.4%) was composed of strains from two different subspecies (Table 4).

**Table 4:** **Concatenated sequence analysis of the seven housekeeping genes clusters**.

**Cluster ID**	**Num./Total**	**% Clustered**	**Serovar/Subspecie**	**Group**	**Shared element of antigenic formula formula**			**O group**
					**O**	**H1**	**H2**	
1	2/4	50	Decatur	I	6,7	c	1,5	O:7
2	2/2	100	Javiana	I	1,9,12	l,z_28_	e,n,z_15_	O:9
3	2/146	1.4		I	#,4,[5],12			
4	2/146	1.4		I	1,9,12		1,5	O:9
5	2/2	100	Montevideo	I	{6,7,14}{54}	g,m,[p],s	[1,2,7]	O:7
6	2/146	1.4		I				
7	3/146	2.1		I	1,4,[5],12		[1,2]	O:4
8	2/11	18.2	Muenchen	I	6,8	D	1,2	O:8
9	2/146	1.4		I				
10	2/3	66.7	Manhattan	I	6,8	D	1,5	O:8
11	7/11	63.6	Muenchen	I	6,8	D	1,2	O:8
12	3/146	2.1		I				
13	2/2	100	Limete	I	1,4,12,27	B	1,5	O:4
14	2/146	1.4		I				
15	2/2	100	Dublin	I	1,9,12[Vi]	g,p	–	O:9
16	5/146	3.4		I	1,9,12		–	O:9
17	4/24	16.7	Paratyphi B	I	1,4,[5],12	B	1,2	O:4
18	2/3	66.7	Newport	I	6,8,20	e,h	1,2	O:8
19	3/13	23.1	Heidelberg	I	1,4,[5],12	R	1,2	O:4
20	2/146	1.4		I	1,4,[5],12		1,2	O:4
21	2/13	15.4	Heidelberg	I	1,4,[5],12	R	1,2	O:4
22	8/146	5.5		I	1,4,[5],12		1,2	O:4
23	3/146	2.1		I				
24	2/24	8.3	Paratyphi B	I	1,4,[5],12	B	1,2	O:4
25	5/10	50	Saintpaul	I	1,4,[5],12	e,h	1,2	O:4
26	2/146	1.4		I	1,4,[5],12		1,2	O:4
27	3/146	2.1		I	6,8	D		O:8
28	2/27	7.4	Typhimurium	I	1,4,[5],12	I	1,2	O:4
29	22/27	81.5	Typhimurium	I	1,4,[5],12	I	1,2	O:4
30	14/24	58.3	Paratyphi B	I	1,4,[5],12	B	1,2	O:4
31	2/2	100	Typhi	I	9,12[Vi]	D	–	O:9
32	2/2	100	Choleraesuis	I	6,7	C	1,5	O:7
33	2/2	100	Paratyphi C	I	6,7[Vi]	C	1,5	O:7
34	2/4	50	*houtenae*	IV-VII	40		–	O:40
35	2/24	8.3	Paratyphi B	I	1,4,[5],12	B	1,2	O:4
36	3/146	2.1		I	1,4,[5],12		1,2	O:4
37	2/2	100	*arizonae*	IIIa	62		–	O:62
38	2/2	100	*salamae*	II				
39	2/2	100	*diarizonae*	IIIb				
40	2/160	1.3	Mix		45			O:45
41	2/2	100	*bongori*	V		z_41_	–	

*^#^extra element not shared by members of this cluster*.

*^_^underlined is to indicate the presence of an O factor due to phage conversion*.

*^{}^O factors within curly brackets indicate that factors in curly brackets cannot coexist with others factors in curly brackets*.

*^[]^O or H factor that may be present or absent without relation to phage conversion*.

**Figure 7:**
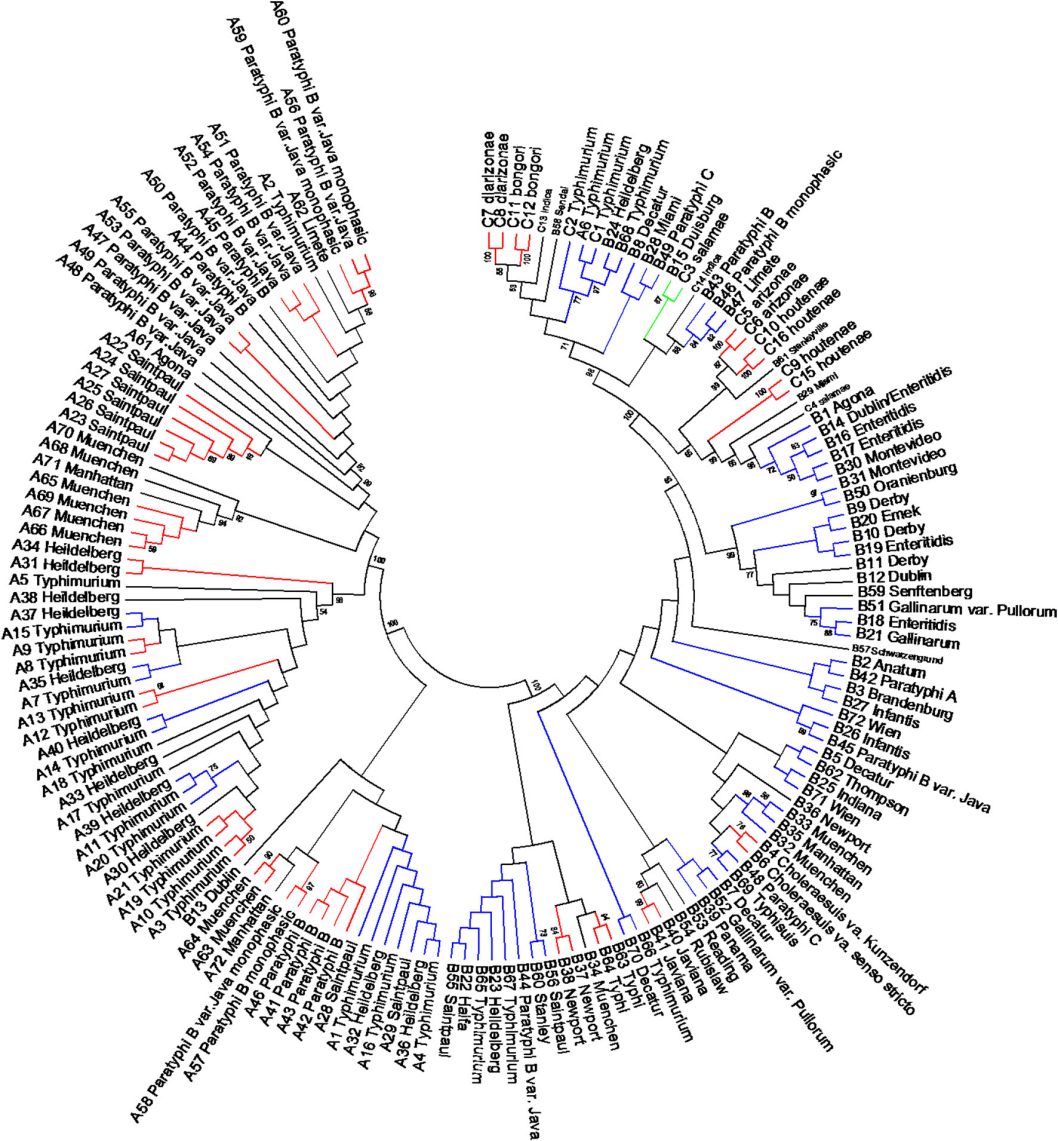
**Concatenated *fliC*, *gnd* and *mutS* genes partial sequences evolutionary relationships of taxa**. The evolutionary history was inferred using the Neighbor-Joining method (Saitou and Nei, 1987). The bootstrap consensus tree inferred from 1000 replicates is taken to represent the evolutionary history of the taxa analyzed (Felsenstein, 1985). Branches corresponding to partitions reproduced in less than 50% bootstrap replicates are collapsed. The evolutionary distances were computed using the Maximum Composite Likelihood method (Tamura et al., 2004) and are in the units of the number of base substitutions per site. The analysis involved 160 nucleotide sequences. All positions containing gaps and missing data were eliminated. There were a total of 1047 positions in the final dataset. Evolutionary analyses were conducted in MEGA5 (Tamura et al., 2011). Specific, Type I and mix clusters are represented in red, blue and green, respectively.

Concatenated analysis of three RFLP genes showed the following. The length of the concatenated *fliC*, *gnd*, and *mutS* genes was 3,907 bp. The number of variable nucleotides was 73.5% among the 160 *Salmonella* species, subspecies, and serovars. Evolutionary analysis, history and distances were determined as for the housekeeping genes in MEGA5 (Saitou and Nei, 1987; Tamura et al., 2004, 2011) (Figure 7). Forty-four different clusters and 34 single serovars were identified for a total of 78 different types (Figure 7). This cluster distribution corresponds to a DP of 0.988. The 44 clusters and the relationship among strains on each cluster are described in Table 5. Twenty-two out of 44 (50.0%) clusters consisted of different homogeneous serovar groups. Thirteen out of the 28 (46.4%) serovars and/or subspecies represented by more than one strain were grouped in homogeneous clusters (Table 5). In six out of these third-teen (46.1%) homogeneous clusters representing serovars and subspecies containing more than one strain, 100% of the representing strains were grouped together. Twenty-one out of 44 (47.7 %) clusters were defined as Type I clusters consisting of *S. enterica* subsp. *enterica* strains. Six (28.6%), four (19.0%) and ten (47.6%) of the 21 Type I clusters did not share, shared one or two elements in their antigenic formula, respectively. Three out of four (75%) clusters shared only the O antigen. Nine out of 10 (90.0%) clusters sharing 2 elements, shared the O and the H2 antigen. Cluster 20 (4.8%) is a Type I cluster composed of two members that shared the H1 and H2 flagellar antigens and differed in one element of the O antigen. Only one cluster out of 44 (2.7%) was composed of strains from two different subspecies but shared the H1 antigen (Table 5).

**Table 5:** **Concatenated sequence analysis of *fliC, gnd* and *mutS* genes clusters**.

**Cluster ID**	**Num./Total**	**% Clustered**	**Serovar/Subspecie**	**Group**	**Shared element of antigenic formula formula**			**O group**
					**O**	**H1**	**H2**	
1	2/2	100	*diarizonae*	IIIb				
2	2/2	100	*bongori*	V				
3	5/27	18.5	Typhimurium	I	1,4,[5],12	i	1,2	O:4
4	3/146	2.1		I			1,5	
5	2/160	1.3	Mix			d		
6	3/146	2.1		I	1,4,#,12,#	b		O:4
7	2/2	100	*arizonae*	IIIa	62			O:62
8	2/4	50	*houtenae*	IV			–	
9	2/4	50	*houtenae*	IV		g,z_51_	–	
10	5/146	3.4		I				
11	2/146	1.4		I	6,7,14,{#}			
12	4/146	2.7		I				
13	3/146	2.1		I	1,9,12		–	O:9
14	4/146	2.7		I				
15	3/146	2.1		I				
16	2/146	1.4		I	6,7,#		1,5	O:7
17	2/146	1.4		I	1,4,12,#			O:4
18	3/146	2.1		I	6,8	d		O:8
19	2/2	100	Choleraesuis	I	6,7	c	1,5	O:7
20	2/146	1.4		I	6,7 [#]	c	1,5	O:7
21	3/146	2.1		I				
22	2/2	100	Javiana	I	1,9,12	l,z_28_	e,n,z_15_	O:9
23	2/146	1.4		I				
24	2/2	100	Typhi	I	9,12[Vi]	d	–	O:9
25	2/3	66.7	Newport	I	6,8,20	e,h	1,2	O:8
26	2/146	1.4		I	1,4,[5],12,#		1,2	O:4
27	6/146	4.1		I	1,4,[5],12		1,2	O:4
28	7/146	4.8		I	1,4,[5],12			O:4
29	4/24	16.7	Paratyphi B	I	1,4,[5],12	b	1,2	O:4
30	2/24	8.3	Paratyphi B	I	1,4,[5],12	b	1,2	O:4
31	2/11	18.2	Muenchen	I	6,8	d	1,2	O:8
32	4/27	14.8	Typhimurium	I	1,4,[5],12	i	1,2	O:4
33	3/146	2.1		I	1,4,[5],12		1,2	O:4
34	2/146	1.4		I	1,4,[5],12		1,2	O:4
35	2/27	7.4	Typhimurium	I	1,4,[5],12	i	1,2	O:4
36	2/146	1.4		I	1,4,[5],12		1,2	O:4
37	2/27	7.4	Typhimurium	I	1,4,[5],12	i	1,2	O:4
38	2/146	1.4		I	1,4,[5],12		1,2	O:4
39	2/13	15.4	Heidelberg	I	1,4,[5],12	r	1,2	O:4
40	4/11	36.4	Muenchen	I	6,8	d	1,2	O:8
41	6/10	60	Saintpaul	I	1,4,[5],12	e,h	1,2	O:4
42	2/24	8.3	Paratyphi B	I	1,4,[5],12	b	1,2	O:4
43	3/24	12.5	Paratyphi B	I	1,4,[5],12	b	1,2	O:4
44	3/24	12.5	Paratyphi B	I	1,4,[5],12	b	1,2	O:4

*^#^Extra element not shared by members of this cluster*.

*^_^underlined is to indicate the presence of an O factor due to phage conversion*.

*^{}^O factors within curly brackets indicate that factors in curly brackets cannot coexist with others factors in curly brackets*.

*^[]^O or H factor that may be present or absent without relation to phage conversion*.

### Multilocus sequence type (st) vs. PCR-RFLP restriction type (RT)

MLST assigns an independent allele number based on sequence differences to each of the seven housekeeping genes. The combination of alleles defines an individual strain multilocus sequence type (ST) (Maiden et al., 1998). To test a different approach in the analysis of PCR-RFLP data collected we assigned a numerical identifier to each of the 930 restriction patterns generated by the restriction digestion of *fliC*, *gnd*, and *mutS* genes PCR products and investigated the relatedness of the 160 *Salmonella* strains by assigning a restriction type (RT). To assign the restriction patterns numbers we took in consideration the following: number of bands, differences in fragments sizes and presence and/or absence of a fragment (Van Belkum et al., 2007). Based on that, we identified 71 and 39 different restriction patterns by cutting the *fliC* gene with *HhaI* and *Sau3AI*, respectively. In the case of the *gnd* gene, we found 39 and 23 different restriction patterns by cutting with *AciI* and *AluI*, respectively. For the *mutS* gene, 41 and 40 different restriction patterns were assigned after digesting the PCR product with *AciI* and *HaeII*, respectively.

To assign the numerical RT we used the same restriction patterns that best clustered the different species, subspecies, and serovars (Figure 5). We assigned 128 different RTs vs. 87 different STs among the 160 strains studied (Tables S4, S5). Among the species, subspecies and serovars with more than one representative (28 possible clusters), we identified in eleven out of 28 (39.3%) the same number of RTs and STs. In four out of the 28 possible homogeneous clusters (14.3%) no STs were identified in the database. These strains belong to *S. bongori*, and *S. enterica* subspecies *arizonae*, *houtenae* and *salamae*. In third-teen out of 28 (46.4%) a higher number of RTs than STs were assigned. Higher diversity in the number of RTs increased with the number of representative in a specific *Salmonella* serovar. In the case of *S*. Typhimurium, Paratyphi B, Heidelberg, Muenchen and Saintpaul, 15, 12, 8, 7, and 10 different RTs were assigned vs. 4,7,2,5, and 4 STs, respectively (Tables S4, S5). To compare and illustrate the clonal structure of *S*. Typhimurium, Paratyphi B, Heidelberg, Muenchen and Saintpaul derived from STs and RTs we used eBURST program (Feil et al., 2004). Results are summarized in Table 6. Given the fact that more RTs were assigned as compared to STs, a more complex clonal structure is observed in all five serotypes using the RTs (Table 6). *S*. Typhimurium clonal structure using the STs consisted of a founder ST 19 containing the majority of the strains (20 out 26, 77%), and connecting two single locus variable STs, 98 and 99, and a singleton ST 36. The *S*. Typhimurium clonal structure based on RTs is composed of one founder RT 6 connecting two subgroup founders RTs 9 and 38; and 3 singletons RTs 33, 92 and 93. Subgroup founder RT 9 is diversified by a third subgroup founder 69 (Table 6). It is interesting to mention that based on ST only singletons STs were observed in *S*. Muenchen. However, using RTs three different clonal groups were identified (Table 6). The typeability of the RT approach when compared to cluster and concatenated sequence analysis of *fliC*, *gnd* and *mutS* was superior (Table S6). We tried to determine epidemiology concordance in respect to the source of isolation. Even though some RTs were unique to the source of isolation for some *Salmonella* strains, the strain specific information was limited to reach a conclusion (Table S7).

**Table 6:** **Comparison of eBURST diagrams of selected *Salmonella enterica* subsp. *enterica* serovars**.

***Salmonella* serovar**	**Num. strains**	**Typing technique**	
		**MLST**	**PCR-RFLP**
Typhimurium	27	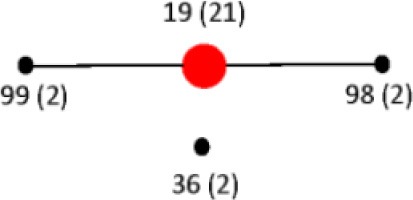	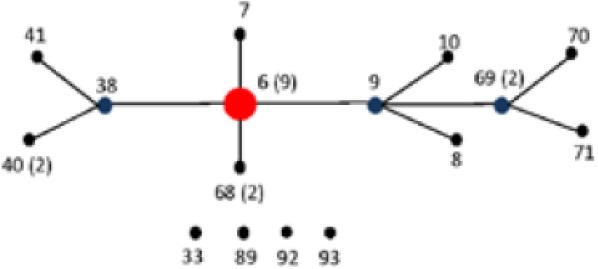
Paratyphi B	24	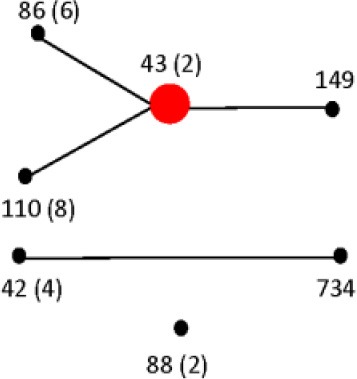	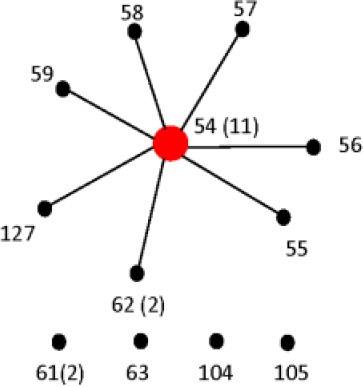
Heidelberg	13	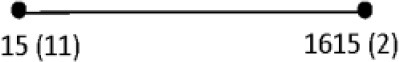	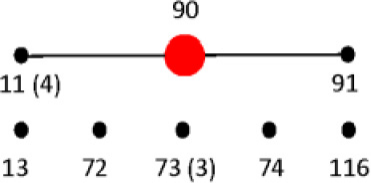
Muenchen	11	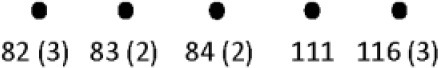	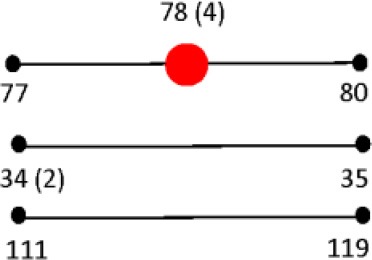
Saintpaul	10	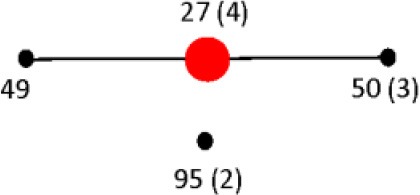	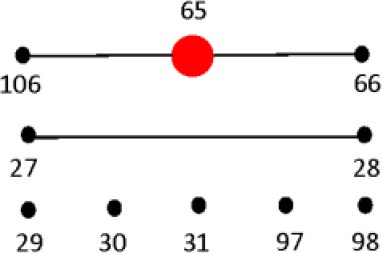

## Discussion

In order to accelerate the process of conventional serotyping researchers have developed and evaluated various molecular methods and combination techniques such as microarrays, sequencing of housekeeping and antimicrobial resistance genes, and whole genome sequence in an attempt to improve the ability to differentiate not only between *Salmonella* serovars, but also between different strains of the same serovar (Porwollik et al., 2004; Grimont and Weill, 2007; Franklin et al., 2011; Braun et al., 2012; Allard et al., 2013; Ranieri et al., 2013). Several methods have been applied for epidemiological studies. Phage typing and MLST have been used for epidemiological studies with limitations (Zheng et al., 2014). MLST can be used for epidemiological studies of any bacterial pathogen that exhibits variability in its housekeeping gene sequences. As an example, in a global collection of *S*. Typhi isolates, only three polymorphic sites were identified among the seven housekeeping genes, partitioning the isolates into four STs (Kidgell et al., 2002).

Here we describe the development and applicability of a typing method for differentiation of *Salmonella* species, subspecies and serovars based on a three genes PCR-RFLP using the *fliC*, *gnd*, and *mutS* genes as targets and the incorporation of the 2100 Agilent Bioanalyzer to facilitate data collection for further analysis. One caveat worth noting in our study was the selection of several genes which are known to have been subject to substantial reticulate evolutionary change in the form of horizontal gene transfer (Nelson and Selander, 1994; Thampapillai et al., 1994; Brown et al., 2002, 2003). Even though that the *gnd* is located in highly variable region, the rate of recombination due to horizontally transferred *gnd* sequences is only moderately higher than the rates for other chromosomal housekeeping genes. This is in contrast with *E. coli* in which several studies of nucleotide sequence variation in *gnd* have identified interstrain transfer and recombination as a factor contributing to an unusually high level of alleles (Nelson and Selander, 1994; Thampapillai et al., 1994). Previous studies have suggested that the *Salmonella mutS* gene has gone intragenic recombination. These recombination events have been however restricted among members of *Salmonella enterica* subspecies I and more limited beyond the other subspecies (Brown et al., 2002, 2003).

While numerous studies have noted extensive recombination among *mutS* and *gnd* across subspecies I *Salmonellae*, it is important to recall the context in which evidence for this allelic shuffling was documented. Evidence for lateral transfer was noted across disparate serovars and, in several cases, across *Salmonella* subspecies (Brown et al., 2002, 2003). However, these changes likely accrued across evolution time during the radiation of *S. enterica*. For molecular epidemiologic utility in real time, the polymorphism itself is what is useful in delimiting the relatedness of outbreak strains and tracing back to an outbreak's source, thus more rapidly evolving markers can often be useful in this regard. Albeit, for evolutionary divergence over a longer time, changes that occur too often could easily obscure phylogenetic relationships. Restated, in this particular instance, the conserved nature of *S. enterica* genomes among closely related serovars confounded an effective differentiation of these serovars, making it difficult to find phylogenetic characters that have undergone change. *mutS* and *gnd* both retained sufficient genetic changes for molecular epidemiological purposes and these changes are not beholden to a rigid evolutionary model for their purposes here (Zheng et al., 2014).

First, we were able to differentiate the two species of *Salmonella* by conventional PCR based on the lack of the PCR amplification of the *gnd* gene. All (8 out of 8 samples) of the *S. bongori* strains tested were PCR negative for the *gnd* gene and positive for the *fliC* and the *mutS* genes under our PCR conditions (Figure 2). We know that the *gnd* gene is present in *S. bongori*, however we determined that the lack of *gnd* amplification was due to a nucleotide mismatch at the 3′-end of the *gnd* F-1 primer (**Figure S1**) (Beard et al., 2004; Johnson and Beese, 2004; Stadhouders et al., 2010). This mismatch provided the opportunity to differentiate *S. enterica* from *S. bongori*, and as far as we know this is a first report of a simple test to differentiate the two *Salmonella* species. In contrast, with the exception of a *S*. Newport strain (SAR B37) that was negative for the *mutS* gene, all the remaining strains tested were PCR positive for the three RFLP target genes.

*Salmonellae* consist of six subspecies. A multiplex PCR assay for *Salmonella* subspecies identification has been published before (Lee et al., 2009). This assay consisted in the PCR amplification of six different target genes and the differentiation was based on the patterns generated by the positive or negative PCR amplification of the selected markers. Fifty of the 53 *Salmonella* strains (94.3%) shown a unique band pattern. Although PCR is a simple test, multiplex PCR can be tricky to apply. By cluster analysis we found that the restriction patterns generated cutting the *mutS* gene PCR product with the *AciI* restriction enzyme has the sufficient potential sufficient to differentiate the six *Salmonella* subspecies into homogeneous clusters (Figure 4). One hundred and forty-six out 160 strains tested in this study belonged to *S. enterica* subsp. *enterica* (Group I) and as a consequence six different homogeneous clusters were identified.

Forty-one different *S. enterica* subsp. *enterica* serovars forming subsp. I and the five remaining subspecies were represented among the 160 strains tested. The best cluster differentiation among *S. enterica* subsp. *enterica* serovars and the five remaining subspecies was achieved by combining restriction patterns obtained from the *fliC* gene cut with *HhaI* and *Sau3AI*; *gnd* gene cut with *AciI* and *AluI*; and the *mutS* gene cut *HaeII*. This enabled us to group 19 out of 28 different *S. enterica* subsp. *enterica* serovars and subspecies into homogeneous clusters (Figure 5 and Table 2). While validating these results will require a larger number of different serovars and strains, we have demonstrated the potential of this technique in the identification of *Salmonella* by its ability to identify *S*. Saintpaul and *S*. Typhimurium in artificially inoculated jalapeño peppers and tomatoes, respectively. Amplification of the *fliC*, *gnd*, and *mutS* genes from DNA extracted from alfalfa sprouts pre-enrichment was not successful. Low yields of PCR product were observed, however *S*. Newport, the serovar used during artificial inoculation of alfalfa sprouts, was identified using the *Salmonella* standard molecular serotyping method (Fitzgerald et al., 2007; Mcquiston et al., 2011). PCR-RFLP has been attempted before for the identification of *Salmonella* strains in cantaloupe and chile peppers production systems in Mexico (Gallegos-Robles et al., 2008) and in shellfish (Albarnaz et al., 2007). Contrary to our study, in which the identification and serotyping was done directly from the 24 h pre-enrichments of jalapeño peppers and tomatoes, these previous studies used pure culture isolates from collected samples and compared to reference *Salmonella* strains (Albarnaz et al., 2007; Gallegos-Robles et al., 2008).

In the present study, we compared the three genes PCR-RFLP cluster analysis, concatenated sequence analysis of the MLST housekeeping genes (Figure 6) and partial sequences of *fliC*, *gnd*, and *mutS* genes (Figure 7) of the 160 strains representing the 2, 6, and 41 *Salmonella* species, subspecies, and serovars, respectively. Discriminatory power was higher in the two sequence based type of analyses (Table S6). However, the simplicity of our PCR-RFLP and its direct application to food samples increases its future potential use. MLST has the advantage of providing unambiguous results because DNA sequences, rather than banding patterns, are analyzed (Maiden et al., 1998). Sequence types are easy to compare between laboratories. Unlike serotyping, MLST recognizes evolutionary groupings and recently has been recommended that *Salmonella* classification by serotyping should be replaced by this technique or its equivalents (Achtman et al., 2012). However, having the same multilocus sequence type does not mean that the strains are genetically identical, because given the fact that only a tiny fraction of the genome is sequenced. For every strain analyzed, seven gene fragments must be sequenced in both directions for a total of 14 sequences. Although sequencing services are becoming more available, the analysis of the sequences can be time-consuming. Simpler and more economical phylogenetic schemes with high discriminative power that are free of recombination bias are preferable. As an example, the complete sequence of the *rpoB* genes were used for the serotyping of 100 *Salmonella* strains representing 40 serovars (Seong et al., 2012). In addition, Seong et al. (2012) introduced the concept of 60 *rpoB* sequence type (RSTs) identifier based on nucleotide differences among test strains gene sequences when compared with an *rpoB* consensus sequence. Phylogenetic analysis showed 60 different RSTs. MLST in the same group of strains identified 49 different ST.

We explored a different approach to analyze the collected RFLP data. Here we introduce the concept of restriction type (RT). A similar concept known as RFLP type was described by Hathaway et al. (2007) for *Streptcoccus pneumoniae*. In their case, numeric IDs were assigned to each one of multiple restriction patterns. However, all the restriction patterns generated came from only one region in the genome of *S*. *pneumonie*. Our RT concept can be considered a hybrid among RFLP and MLST. In our case we assigned numeric IDs to each one of the different restriction patterns as described before (Hathaway et al., 2007) however we used three different regions of the *Salmonella* genome, the *fliC*, *gnd*, and *mutS* genes. The combination of five out the six restriction patterns among the three genes formed a unique strain ID or RT. Based on that we were able to assigned a total of 128 unique RTs (Tables S4, S6). When compared to MLST, 81 STs were identified among the same strains. All the RTs identified were specific for a given serotype demonstrating a higher typeability when compared to the any other of the clusters or concatenated sequence analysis tested (Tables S4, S6).

Our current study is the first research adapting PCR-RFLP for *Salmonella* molecular typing using the 2100 Agilent 2100 Bioanalyzer. The Agilent 2100 Bioanalyzer is relatively inexpensive (~$24,000.00) and simple to operate, compared to other commercially available capillary electrophoresis devices. Analysis with the Agilent 2100 Bioanalyzer yields several important advantages compared to traditional separation, imaging, and analysis techniques. Due to its sensitivity 1 μl of sample is required for the analysis of nucleic acids in real time. Results of the resolved nucleic acids are delivered within 30 min. Prepackaged kits, standardized sample preparation and automated analysis yield more accurate and reproducible data due to decreased manual intervention. These characteristics allows the comparative analysis of data obtained at different dates. Overall, the Agilent 2100 Bioanalyzer allows analysis of DNA fragments, including chip preparation, separation, detection and data analysis to be done in a shorter period of time when compared to other serotyping methods.

The cost of subgrouping and serotyping *Salmonella* using the three genes PCR-RFLP per sample ranges from $12.25 (using NEB restriction enzymes) to $19.28 (using Fermentas restriction enzymes). The use of NEB restriction enzymes will decrease the cost per sample but will increase the time of processing due to the additional hour required for restriction digestion of the DNA. In contrast, typing techniques such as MLST has been estimated to cost closer to $35.00 per sample, and between $35.00 and $135.00 per isolate for traditional serotyping (Achtman et al., 2012; Guard et al., 2012). Given the frequency of *Salmonella* outbreaks, these cost savings could become significant over time.

## Conclusions

Previous *Salmonella* RFLP studies and those using the Agilent 2100 Bioanalyzer have been concentrated in one area of the genome. Our method increases the discriminatory power of RFLP by using three genes (*fliC*, *gnd*, and *mutS*) and digesting each gene with two enzymes. The combination of five out the six restriction patterns generated digesting the *fliC*, *gnd*, and *mutS* genes showed a good discriminatory power by cluster analysis but it was superior using the RTs. While PCR amplification of the *fliC*, *gnd*, and *mutS* genes appears to be affected by the type of food commodity tested, we believe our method of PCR-RFLP may be a cost-effective tool for narrowing down the number of possible *Salmonella* serovars in 24-hour pre-enrichment samples. Contrary to conventional agarose gels, the sensitivity of the bioanalyzer can be adjusted to increasing its capacity of detection of the fragments. Co-migrating bands are reported by this device facilitating the determination of complete restriction digestion. The report of the molar concentration of the restriction fragments allows the use stoichiometric distribution as an indicator of complete digestion under the conditions tested. In addition, different runs from different dates can be compared facilitating the normalization and identification of different restriction patterns. The incorporation of automation in PCR-RFLP will facilitate the creation of databases that can be compared between laboratories following a standard procedure describing the preparation, processing, and analysis of the samples.

## Author contributions

Ángel A. Soler-García and Eric W. Brown contributed with the conception and design of the study. Ángel A. Soler-García, Antonio J. De Jesús, and Kishana Taylor were involved in the collection and assembly of data. Ángel A. Soler-García, Antonio J. De Jesús, Kishana Taylor, and Eric W. Brown were involved in the analysis and interpretation of data; and drafting of the article, critical revision of the article for important intellectual content and final approval of the article.

### Conflict of interest statement

The authors declare that the research was conducted in the absence of any commercial or financial relationships that could be construed as a potential conflict of interest.
