# Supplementary material for: Differentiation of Salmonella strains from the SARA, SARB and SARC reference collections by using three genes PCR-RFLP and the 2100 Agilent Bioanalyzer
Source: Front Microbiol. 2014 Aug 11;5:417. doi: 10.3389/fmicb.2014.00417 (PMC4127528; doi:10.3389/fmicb.2014.00417)
Supplement: Supplementary file 1 [file DataSheet1.DOC]

**Supplementary Table 1. Conventional, qPCR, sequencing primers and probes**

| **Primers**  **and Probes** | **Sequence** | **Gene** | **Amplicon**  **(bp)** | **Analyzed**  **Nucleotides**  **(bp)** | **Reference** |
| --- | --- | --- | --- | --- | --- |
| *fliC-*F  *fliC-*R  *gnd*-F  *gnd*-R  *SMNH*-F  *MSCT*-R  aroC-F  aroC-R  dnaN-F  dnaN-R1  hemD-F  hemD-F1  hemD-R  hisD-F  hisD-R  purE-F  purE-R  purE-R1  sucA-F  sucA-R  thrA-F  thrA-R  thrA-R1  invA-176F  invA-291R  invA-Tx-208  IAC-F  IAC-R  IAC-Cy5 | 5’-GCACAAGTCATTAATACAAACAGCC-3’  5’-TTAACGCAGTAAAGAGAGGACG-3’  5’-CTGCGCCTGAATTAAGTTAGCTGG-3’  5’-GAAAGCCGTGGTTATACCGTCTCC-3’  5’-CCGGGCTGGATACGCTGAAAG-3’  5’-TTCAGCCGATAGATCCACTCCA-3’  5’-CCCAGTCACGACGTTGTAAAACGACTGGCACCTCGCGCTATAC-3’  5’- AGCGGATAACAATTTCACACAGGAACCACACACGGATCGTGGCG-3’  5’-CCCAGTCACGACGTTGTAAAACGATGAAATTTACCGTTGAACGTGA-3’  5’-AGCGGATAACAATTTCACACAGGAACCGCGGAATTTCTCATTCGAG-3’  5’-CCCAGTCACGACGTTGTAAAACGATGAGTATTCTGATCACCCG-3’  5’CCCAGTCACGACGTTGTAAAACGGAAGCGTTAGTGAGCCGTCTGCG-3’  5’-AGCGGATAACAATTTCACACAGGAAATCAGCGACCTTAATATCTTGCCA-3’  5’-CCCAGTCACGACGTTGTAAAACGGAAACGTTCCATTCCGCGCAGAC-3'  5’-AGCGGATAACAATTTCACACAGGAACTGAACGGTCATCCGTTTCTG-3’  5’-CCCAGTCACGACGTTGTAAAACGATGTCTTCCCGCAATAATCC-3’  5’-AGCGGATAACAATTTCACACAGGAATCATAGCGTCCCCCGCGGATC-3’  5’-AGCGGATAACAATTTCACACAGGAACGAGAACGCAAACTTGCTTC-3’  5’- CCAGTCACGACGTTGTAAAACGAGCACCGAAGAGAAACGCTG-3’  5’-AGCGGATAACAATTTCACACAGGAAGGTTGTTGATAACGATACGTAC-3’  5’-CCCAGTCACGACGTTGTAAAACGGTCACGGTGATCGATCCGGT-3’  5’-AGCGGATAACAATTTCACACAGGAACACGATATTGATATTAGCCCG-3’  5’-AGCGGATAACAATTTCACACAGGAAGTGCGCATACCGTCGCCGAC-3’  5′-CAACGTTTCCTGCGGTACTGT-3′  5′-CCCGAACGTGGCGATAATT-3′  5′-TX-CTCTTTCGTCTGGCATTATCGATCAGTACCA-BHQ2-3′  5′-CTAACCTTCGTGATGAGCAATCG-3′  5′-GATCAGCTACGTGAGGTCCTAC-3′  5′-Cy5-AGCTAGTCGATGCACTCCAGTCCTCCT-Iowa Black RQ-Sp-3′ | *fliC*  *gnd*  *mutS*  *aroC*  *dnaN*  *hemD*  *hisD*  *purE*  *sucA*  *thrA*  *invA*  IAC | 1,515  1,266  1,176  826  833  666  894  510  643  852  116  198 | 1,324  1,050  1,019  480  501  432  501  345  435  444 | Gallegos-Robles et al., 2008  This study  Brown et al., 2002  Deer et al., 2010  Deer et al., 2010 |

*More details of the primers used to amplify and sequence the seven housekeeping for MLST can be found at the MLST Databases at the ERI, University College Cork (http://mlst.ucc.ie/mlst/dbs/Senterica).
