# Supplementary material for: Differentiation of Salmonella strains from the SARA, SARB and SARC reference collections by using three genes PCR-RFLP and the 2100 Agilent Bioanalyzer
Source: Front Microbiol. 2014 Aug 11;5:417. doi: 10.3389/fmicb.2014.00417 (PMC4127528; doi:10.3389/fmicb.2014.00417)
Supplement: Supplementary file 2 [file DataSheet2.DOC]

**Supplementary Table 2.** Exclusivity test for the specificity of *fliC*, *gnd*, and *mutS* genes PCR amplification.

| SAFE  ID | Bacteria | Original  ID | Amplified gene | | |
| --- | --- | --- | --- | --- | --- |
|  |  |  | *fliC* | *gnd* | *mutS* |
| 102 | *Vibrio cholerae* | 11629 | - | - | - |
| 103 | *Vibrio metschnikovii* | 7708 | - | - | - |
| 104 | *Vibrio parahaemolyticus* | 17802 | - | - | - |
| 105 | *Vibrio vulnificus* | 29306 | - | - | - |
| 106 | *Escherichia coli* | EC P1334 | - | - | +* |
| 107 | *Escherichia coli* | EC CL-15 | - | - | - |
| 108 | *Shigella sonnei* | SHI0168 | - | - | +* |
| 109 | *Shigella flexneri* | 670 | - | - | +* |
| 110 | *Shigella dysenteriae* | 973 | - | - | +* |
| 111 | *Shigella boydii* | 970 | - | - | - |
| 112 | *Proteus vulgaris* | 66N | - | - | - |
| 113 | *Klebsiella pneumoniae* | NES14 | - | - | - |
| 114 | *Bacillus cereus* | 6A16 | - | - | - |
| 115 | *Bacillus subtilis* | 3A16 | - | - | - |
| 116 | *Citrobacter Freundii* | 47N | - | - | +* |
| 117 | *Erwinia mallotivora* | 8645 | - | - | - |
| 118 | *Brenneria nigrifluens* | 1391 | - | - | - |
| 119 | *Cronobacter sakazaki* | E604 | - | - | - |
| 120 | *Cronobacter malonaticus* | E265 | - | - | - |
| 121 | *Cronobacter dublinensis* | E464 | - | - | - |

* Low yield of PCR product as compared to *Salmonella* positive control*.*
