# Supplementary material for: Differentiation of Salmonella strains from the SARA, SARB and SARC reference collections by using three genes PCR-RFLP and the 2100 Agilent Bioanalyzer
Source: Front Microbiol. 2014 Aug 11;5:417. doi: 10.3389/fmicb.2014.00417 (PMC4127528; doi:10.3389/fmicb.2014.00417)
Supplement: Supplementary file 3 [file DataSheet3.DOC]

**Supplementary Table 3.** Comparison of restriction patterns from published *Salmonella* genomes and experimental data

| *Salmonella* (ID) | Parameters | Gene | | | | | | | | | | | |
| --- | --- | --- | --- | --- | --- | --- | --- | --- | --- | --- | --- | --- | --- |
|  |  | *fliC* | | | | *gnd* | | | | *mutS* | | | |
|  |  | *Hha*I | | *Sau3A*I | | *Aci*I | | *Alu*I | | *A*ciI | | *Hae*II | |
|  |  | *In*  *silico* | Exp. | *In*  *silico* | Exp. | *In*  *silico* | Exp. | *In*  *silico* | Exp. | *In*  *silico* | Exp. | *In*  *silico* | Exp. |
| Typhimurium  (A2) | Num. bands  Rest. Fragments (bp)  % difference | 682  306  116  113  104  61  47  36  16  2  2  11  1,485 | 318  224*  145  122  118  109  67*  9  1,394  6.1 | 700  475  299  11  4  1,485 | 1146  474  2  1,620  8.3 | 189  153  147  132  127  79  73  67  59  56  50  36  35  32  31  15  1,266 | 192  146*  130  122  81  74  68  58*  51  33*  15  1,258  0.6 | 272  257  243  97  95  84  80  74  23  20  13  8  12  1,266 | 272  256  247  97*  85  79*  8  1,212  4.3 | 204  162  150  112  99  60  52  44  44  40  40  39  33  28  24  15  13  9  6  3  20  1,177 | 165  152  116  99  62*  53*  47  37*  14  1,163  1.2 | 511  184  153  105  84  70  42  22  6  9  1,177 | 554  191  163  113  96  80  6  1,197  1.7 |
| Paratyphi A  (B42) | Num. bands  Rest. Fragments (bp)  % difference | 487  195  146  142  116  113  104  61  47  36  16  12  6  2  2  15  1,485 | 685  146*  123  118  109  68  59  8  1,454  2.1 | 475  388  304  232  75  11  6  1,485 | 482  404  392  162  87  5  1,527  2.8 | 153  147  127  104  85  82  79  73  67  59  56  50  38  36  35  32  31  12  18  1,266 | 150*  125  93  87  83  78  71  62*  52*  32*  24  15  1,153  8.9 | 339  243  239  97  95  84  80  33  23  20  13  11  1,266 | 344  275  251  100*  88  6  1,158  8.5 | 204  162  150  99  93  60  52  44  44  40  40  39  33  28  24  19  15  13  9  6  3  21  1,177 | 208  166  153  117  101  64  54  38*  25  13  1,078  8.4 | 511  184  153  105  84  70  42  22  6  8  1,177 | 579  200*  169  121  102  86  7  1,257  6.3 |
| Paratyphi C  (B49) | Num. bands  Rest. Fragments (bp)  % difference | 622  174  152  142  116  104  78  47  36  16  12  2  2  13  1,503 | 664  172  152  144  122  107  84  7  1,445  3.9 | 1017  475  11  3  1,503 | 1160  478  2  1,638  8.2 | 153  147  130  127  82  79  73  67  59  59  56  50  50  36  35  32  31  17  1,266 | 148*  137  124  86  82  77  70  61*  52  32*  24  14  1,134  10.4 | 419  272  243  97  95  84  23  20  23  9  1,266 | 410  273  248  97*  84  6  1,209  4.5 | 204  162  150  93  78  60  52  44  44  40  40  39  33  28  24  21  19  15  13  9  6  3  22  1,177 | 207  165  152  95  80  63  53  39*  13  1,088  7.6 | 511  254  153  105  84  42  22  6  8  1,177 | 581  266  166  118  100  5  1,231  4.3 |
| *S. arizonae*  (C5) | Num. bands  Rest. Fragments (bp)  % difference | 676  160  116  104  78  47  37  36  2  10  1,272 | 629  164  122  109  81  57  41*  8  1,244  2.2 | 375  322  266  126  117  39  27  7  1,272 | 367  327  275  126  120  44  23*  13  1,420  10.4 | NR  NR | 240  204  189  142  125  70  61*  47  33*  24  13  1,262  100.0 | NR  NR | 617  272  247  94  4  1,230  100.0 | NR  NR | 205  165  154  116  81  63  54  42*  25  22*  15  1,117  100.0 | NR  NR | 822  157  115  93  4  1,187  100.0 |

* co-migrating bands; NR, no restriction fragments due to negative virtual PCR.
