# Supplementary material for: Differentiation of Salmonella strains from the SARA, SARB and SARC reference collections by using three genes PCR-RFLP and the 2100 Agilent Bioanalyzer
Source: Front Microbiol. 2014 Aug 11;5:417. doi: 10.3389/fmicb.2014.00417 (PMC4127528; doi:10.3389/fmicb.2014.00417)
Supplement: Supplementary file 4 [file DataSheet4.DOC]

**Supplementary Table 4.** Summary of total number of restriction types and multilocus sequence types for each *Salmonella* species, subspecies and serovars.

| Serotype  or  Subgroup | Total | Serotyping Technique | | | | | | | | | | | | | | |
| --- | --- | --- | --- | --- | --- | --- | --- | --- | --- | --- | --- | --- | --- | --- | --- | --- |
|  |  | PCR-RFLP | | | | | | | MLST | | | | | | | |
|  |  | Num. different Restriction Patterns (unique) | | | | | | | Num. of different loci  (unique) | | | | | | | |
|  |  | *fliC* | | *gnd* | | *mutS* | | RT |  |  |  |  |  |  |  |  |
|  |  | *HhaI* | *Sau3AI* | *AciI* | *AluI* | *AciI* | *HaeII* |  | *aroC* | *dnaN* | *hemD* | *hisD* | *purE* | *sucA* | *thrA* | ST |
| Agona  Anatum  *arizonae*  *bongori*  Brandernburg  Choleraesuis  Decatur  Derby  *diarizonae*  Dublin  Dublin/Enteritidis  Duisburg  Emek  Enteritidis  Gallinarum  Haifa  Heidelberg  *houtenae*  Indiana  *indica*  Infantis  Javiana  Limete  Manhattan  Miami  Montevideo  Muenchen  Newport  Oranienburg  Panama  Paratyphi A  Paratyphi B  Paratyphi C  Reading  Rubislaw  Saintpaul  *salamae*  Schwarzengrund  Sendai  Senftenberg  Stanley  Stanleyville  Thompson  Typhi  Typhimurium  Typhisuis  Wien | 2  1  2  2  1  2  4  3  2  2  1  1  1  4  3  1  13  4  1  2  2  2  2  3  2  2  11  3  1  1  1  24  2  1  1  10  2  1  1  1  1  1  1  2  27  1  2 | 2(1)  1  2 (2)  2 (2)  1 (1)  2 (1)  4 (4)  3 (2)  2 (2)  2  1  1 (1)  1  2 (1)  3  1 (1)  4 (1)  4 (4)  1 (1)  2 (1)  2  2 (2)  2 (1)  3 (1)  2 (1)  1  4 (3)  2  1 (1)  1 (1)  1 (1)  3 (2)  2 (1)  1 (1)  1  6 (6)  2 (2)  1 (1)  1  1  1 (1)  1  1 (1)  2 (1)  6 (3)  1  2 (1) | 2  1  2 (2)  2 (1)  1 (1)  2  1  2  2 (1)  2  1  1 (1)  1 (1)  3 (1)  3 (2)  1  2 (1)  2 (2)  1  2 (1)  1  1  1  1  2 (1)  1  1  3  1 (1)  1  1 (1)  1  1  1  1  3 (1)  2 (1)  1  1  1  1  1 (1)  1  2 (1)  2  1  1 | 2  1  2 (2)  -  1 (1)  2  3 (2)  3  2 (2)  1  1  1  1  3  1  1  3 (2)  4 (4)  1  2 (1)  1  2 (1)  2  3 (2)  1  2 (1)  2  3 (1)  1  1  1  4 (1)  2  1  1  4  2 (2)  1  1 (1)  1 (1)  1  1 (1)  1  2  2 (1)  1  2 (1) | 2  1  1 (1)  -  1  2 (1)  2  2  2 (2)  1  1  1  1  3  2  1  2  3 (2)  1  2 (1)  1  2  2  2 (1)  2  1  2  2  1  1  1  2  1  1  1  4 (1)  2 (2)  1  1  1  1  1  1  1 (1)  3 (1)  1  2 | 2  1  1 (1)  1 (1)  1  2 (1)  4 (2)  3  1 (1)  2  1  1  1  3 (1)  1  1 (1)  2  3 (3)  1  2 (2)  2 (1)  2  2 (1)  2  2 (1)  2 (1)  4  2 (1)#  1  1  1  4 (2)  2  1 (1)  1  6 (2)  2 (2)  1  1  1  1  1 (1)  1 (1)  2 (1)  4 (1)  1  2 | 2 (1)  1  1 (1)  1 (1)  1  2  2  2  1  1  1  1  1  2 (1)  1  1  3 (1)  2 (2)  1  1  2 (1)  1  2  2  2  1  2 (1)  3 (1)#  1  1  1  8 (4)  2  1  1 (1)  8 (6)  1 (1)  1  1  1  1 (1)  1  1  2  4 (1)  1 (1)  1 | 2  1  2  2  1  2  4  3  2  2  1  1  1  4  3  1  8  4  1  2  2  2  2  3  2  2  7  3  1  1  1  12  2  1  1  10  2  1  1  1  1  1  1  2  16  1  2 | 1  1  2 (2)  2 (2)  1  2 (2)  3 (3)  3 (3)  2 (2)  1  1 (1)  1  1  3 (1)  1  1  1  3 (3)  1  1 (1)  2 (1)  1 (1)  1 (1)  2 (1)  2 (1)  1  2 (1)  3  1 (1)  1  1 (1)  3 (2)  1  1 (1)  1 (1)  3 (1)  2 (2)  1  1 (1)  1  1  1 (1)  1  1 (1)  4 (2)  1  2 (2) | 1  1  1 (1)  2 (1)  1  1  3 (3)  3 (2)  1 (1)  1  1  1 (1)  1 (1)  3 (1)  1  1  1  3 (3)  1 (1)  2 (2)  2 (1)  1 (1)  1 (1)  2  2 (2)  1 (1)  3 (1)  3 (1)  1  1  1  5 (1)  1  1  1 (1)  4 (1)  1 (1)  1 (1)  1 (1)  1 (1)  1 (1)  1 (1)  1 (1)  1 (1)  2  1  2 (1) | 1 (1)  1  2 (2)  1 (1)  1 (1)  1  3 (2)  2  2 (2)  1  1  1 (1)  1  3 (1)  2 (1)  1  1  3 (3)  1 (1)  2 (2)  2 (1)  1  1 (1)  2 (1)  2  1 (1)  3  3 (1)  1  1  1  4 (2)  1  1  1 (1)  4  1 (1)  1 (1)  1  1 (1)  1 (1)  1  1  1 (1)  3 (1)  1  2 (1) | 1 (1)  1 (1)  2 (2)  2 (2)  1 (1)  1  3 (2)  3 (3)  1 (1)  2 (1)  1  1  1  3 (2)  1  1  2  4 (4)  1 (1)  2 (2)  2 (2)  1 (1)  1 (1)  3 (1)  2 (2)  2 (1)  3 (1)  3 (1)  1 (1)  1  1 (1)  3 (1)  1  1  1 (1)  2  2 (2)  1 (1)  1 (1)  1 (1)  1 (1)  1  1  1 (1)  1  1  2 (2) | 1  1  2 (2)  1 (1)  1  2  3 (1)  2 (1)  1 (1)  2  1  1 (1)  1  3 (1)  1  1  5  3 (3)  1  1 (1)  2 (1)  1 (1)  2 (1)  3 (1)  2 (1)  2 (2)  6  2 (1)  1 (1)  1  1  13 (3)  1  1  1 (1)  5 (1)  2 (2)  1  1 (1)  1 (1)  1 (1)  1 (1)  1 (1)  1 (1)  2  1  2 (2) | 2 (2)  1  2 (2)  1 (1)  1  2 (1)  3 (1)  2 (1)  2 (2)  2 (1)  1  1  1  3 (1)  2 (1)  1  2  2 (2)  1 (1)  2 (2)  2 (1)  1 (1)  2  3  2 (1)  1  4 (1)  3 (1)  1  1  1  4 (1)  2 (2)  1  1  5 (1)  2 (2)  1  1 (1)  1  1  1 (1)  1  1 (1)  3  1(1)  2 (2) | 2 (2)  1 (1)  2 (1)  1 (1)  1  1  3  2 (2)  1 (1)  1 (1)  1  1 (1)  1  3 (1)  1  1  1  3 (3)  1 (1)  2 (2)  2 (2)  1  1 (1)  3  2 (1)  1  4  2 (1)  1  1  1  6 (2)  2 (1)  1  1  5  2 (2)  1  1 (1)  1  1  1 (1)  1 (1)  2 (2)  6 (1)  1 (1)  2 (2) | 1  1  -*  -*  1  2  3  3  2  2  1  1  1  3  2  1  2  1*  1  1*  2  1  1  2  2  2  5  3  1  1  1  7  2  1  1  4  -*  1  1  1  1  1  1  2  4  1  2 |
| Total  Unique | 160 | 71  57 | 39  21 | 39  27 | 23  13 | 41  29 | 40  25 | 128 | 52  42 | 46  36 | 46  32 | 53  46 | 56  35 | 49  35 | 52  36 | 81 |

* No STs were obtained for some of the strains: *S*. *arizonae* (2/2), *S*. *bongori* (2/2), *S*. *houtenae* (3/4), *S*. *indica* (1/2), and *S*. *salamae* (2/2)

# *S*. Newport (B37) was PCR negative for *mutS*.

- *S*. *bongori* was PCR negative for *gnd*.
