# Supplementary material for: Differentiation of Salmonella strains from the SARA, SARB and SARC reference collections by using three genes PCR-RFLP and the 2100 Agilent Bioanalyzer
Source: Front Microbiol. 2014 Aug 11;5:417. doi: 10.3389/fmicb.2014.00417 (PMC4127528; doi:10.3389/fmicb.2014.00417)
Supplement: Supplementary file 5 [file DataSheet5.DOC]

**Supplementary Table 5. *Salmonella* species, subspecies and serovars restriction types.**

| Isolate | Collection  ID | Original  designation | Serotype/  Specie/  Subspecie | Group | Restriction Pattern IDs | | | | | | **RT** |
| --- | --- | --- | --- | --- | --- | --- | --- | --- | --- | --- | --- |
|  |  |  |  |  | *fliC*  *HhaI* | *fliC*  *Sau3AI* | *gnd*  *AciI* | *gnd*  *AluI* | *mutS*  *AciI* | *mutS*  *HaeII* |  |
| 1  2  3  4  5  6  7  8  9  10  11  12  13  14  15  16  17  18  19  20  21  22  23  24  25  26  27  28  29  30  31  32  33  34  35  36  37  38  39  40  41  42  43  44  45  46  47  48  49  50  51  52  53  54  55  56  57  58  59  60  61  62  63  64  65  66  67  68  69  70  71  72  73  74  75  76  77  78  79  80  81  82  83  84  85  86  87  88  89  90  91  92  93  94  95  96  97  98  99  100  101  102  103  104  105  106  107  108  109  110  111  112  113  114  115  116  117  118  119  120  121  122  123  124  125  126  127  128  129  130  131  132  133  134  135  136  137  138  139  140  141  142  143  144  145  146  147  148  149  150  151  152  153  154  155  156  157  158  159  160 | A1  A2  A3  A4  A5  A6  A7  A8  A9  A10  A11  A12  A13  A14  A15  A16  A17  A18  A19  A20  A21  A22  A23  A24  A25  A26  A27  A28  A29  A30  A31  A32  A33  A34  A35  A36  A37  A38  A39  A40  A41  A42  A43  A44  A45  A46  A47  A48  A49  A50  A51  A52  A53  A54  A55  A56  A57  A58  A59  A60  A61  A62  A63  A64  A65  A66  A67  A68  A69  A70  A71  A72  B1  B2  B3  B4  B5  B6  B7  B8  B9  B10  B11  B12  B13  B14  B15  B16  B17  B18  B19  B20  B21  B22  B23  B24  B25  B26  B27  B28  B29  B30  B31  B32  B33  B34  B35  B36  B37  B38  B39  B40  B41  B42  B43  B44  B45  B46  B47  B48  B49  B50  B51  B52  B53  B54  B55  B56  B57  B58  B59  B60  B61  B62  B63  B64  B65  B66  B67  B68  B69  B70  B71  B72  C1  C2*  C3  C4  C5  C6  C7  C8  C9  C10  C11  C12  C13  C14  C15  C16 | INSP 24  LT2  NVSL 7095  NVSL 5820  IVB 232  CDC B1213  IVB 665/81  IVB 5560  NVSL 2816  NVSL 6814  IVB 276/25  NVSL 6993  IVB 1430  IVB 75/67  NVSL 6968  CDC B1236  IVB 48/81  NVSL 6938  INSP 85  IVB 1544  USFW 318  CDC B1605  CDC B1722  CDC B2076  IVB 615  IP 67/88  IP 78/88  IP 86/88  CDC B1400  NVSL 7039  NVSL 5876  NVSL 5145  INSP 94  IVB 7135/1990  IVB 126/82  IVB 588/24  NVSL 5208  NVSL 4960  CDC B2487  IVB 218/82  DMS 155/76  DMS 724/74  DMS 220/82  DMS 2434  IP 7/88  DMS 3254/7/81  DMS 3205/83  DMS 843/82  DMS 2442  DMS 106/76  DMS 53/76  IP 87/87  IP 16/88  IP 8/88  DMS 47/81  DMS 83/76  DMS 2471  DMS 59/81  DMS 61/81  DMS 52/76  DMS 203/74  DMS 53/81  IP 6/88  NVSL 519  NVSL 2817  CDC B2026  INSP 46  IP 15/88  1P 11/88  1P 25/88  CDC B1293  IP 31/88  IVB 36/79  CDC B1487  DMS 2819  NVSL 6321  IVB 651/79  CDC 3327/54  IP 6562/88  IP 631 K  NVSL 4111  NVSL 5558  NVSL 5283  NVSL 5618  IP 82/3144  IVB 3540/24  DMS 3618  CDC SSU7998  IVB 176/82  CDC SSU8074  IVB 470/82  IVB 4793/3366  CDC 4801/72  DMS 3005  NVSL7039  IVB 588/24  DMS 3702  CDC B3460  IVB 385/72  CDC 4648/53  IP 2/79  CDC B2131  CDC B2604  ATCC 8388  IP 11/88  IP 25/88  CDC B1293  CDC B3465  INSP 15  NVSL 3882  IVB Bendia  CDC B1171  CDC B1433  ATCC 9150  DMS 155/76  DMS 106/76  DMS 83/76  DMS 2471  DMS 53/81  IP 33 K  IP 2/88  IP 4/77  IVB 978/87  IVB Italian Standard  DMS 3853  ATCC 10717  CDC B2076  CDC B1400  DMS 1253  CDC 1035/74  NVSL 6673  DMS 1112  DMS 3705  CDC B2637  IP E.88.374  IP E.88.353  INSP 24  NVSL 2816  IVB 1430  USFW 318  CDC 277/68  CDC 1426/67  IP 5/88  IP 3/88  S 6623  IP E.88.374  CDC 151-85  CDC 3472-64  CDC 346-86  CDC 409-85  CDC 156-87  CDC 678-94  CDC 287-86  CDC 750-72  CDC 750-72  CDC 2703-76  CDC 1363-65  CDC 347-78  CDC 2439-64 | Typhimurium  Typhimurium  Typhimurium  Typhimurium  Typhimurium  Typhimurium  Typhimurium  Typhimurium  Typhimurium  Typhimurium  Typhimurium  Typhimurium  Typhimurium  Typhimurium  Typhimurium  Typhimurium  Typhimurium  Typhimurium  Typhimurium  Typhimurium  Typhimurium  Saintpaul  Saintpaul  Saintpaul  Saintpaul  Saintpaul  Saintpaul  Saintpaul  Saintpaul  Heidelberg  Heidelberg  Heidelberg  Heidelberg  Heidelberg  Heidelberg  Heidelberg  Heidelberg  Heidelberg  Heidelberg  Heidelberg  Paratyphi B  Paratyphi B  Paratyphi B  Paratyphi B  Paratyphi B  Paratyphi B  Paratyphi B var. Java  Paratyphi B var. Java  Paratyphi B var. Java  Paratyphi B var. Java  Paratyphi B var. Java  Paratyphi B var. Java  Paratyphi B var. Java  Paratyphi B var. Java  Paratyphi B var. Java  Paratyphi B var. Java  Paratyphi B monophasic  Paratyphi B var. Java monophasic  Paratyphi B var. Java monophasic  Paratyphi B var. Java monophasic  Agona  Limete  Muenchen  Muenchen  Muenchen  Muenchen  Muenchen  Muenchen  Muenchen  Muenchen  Manhattan  Manhattan  Agona  Anatum  Brandenburg  Choleraesuis var. Kunzendorf  Decatur  Choleraesuis var. senso stricto  Decatur  Decatur  Derby  Derby  Derby  Dublin  Dublin  Dublin/Enteritidis  Duisburg  Enteritidis  Enteritidis  Enteritidis  Enteritidis  Emek  Gallinarum  Haifa  Heidelberg  Heidelberg  Indiana  Infantis  Infantis  Miami  Miami  Montevideo  Montevideo  Muenchen  Muenchen  Muenchen  Manhattan  Newport  Newport  Newport  Panama  Javiana  Javiana  Paratyphi A  Paratyphi B  Paratyphi B var. Java  Paratyphi B var. Java  Paratyphi B monophasic  Limete  Paratyphi C  Paratyphi C  Oranienburg  Gallinarum var. Pullorum  Gallinarum var. Pullorum  Reading  Rubislaw  Saintpaul  Saintpaul  Schwarzengrund  Sendai  Senftenberg  Stanley  Stanleyville  Thompson  Typhi  Typhi  Typhimurium  Typhimurium  Typhimurium  Typhimurium  Typhisuis  Decatur  Wien  Wien  Typhimurium  Typhimurium  *salamae*  *salamae*  *arizonae*  *arizonae*  *diarizonae*  *diarizonae*  *houtenae*  *houtenae*  *bongori*  *bongori*  *indica*  *indica*  *houtenae*  *houtenae* | I  I  I  I  I  I  I  I  I  I  I  I  I  I  I  I  I  I  I  I  I  I  I  I  I  I  I  I  I  I  I  I  I  I  I  I  I  I  I  I  I  I  I  I  I  I  I  I  I  I  I  I  I  I  I  I  I  I  I  I  I  I  I  I  I  I  I  I  I  I  I  I  I  I  I  I  I  I  I  I  I  I  I  I  I  I  I  I  I  I  I  I  I  I  I  I  I  I  I  I  I  I  I  I  I  I  I  I  I  I  I  I  I  I  I  I  I  I  I  I  I  I  I  I  I  I  I  I  I  I  I  I  I  I  I  I  I  I  I  I  I  I  I  I  I  I  II  II  IIIa  IIIa  IIIb  IIIb  IV  IV  V  V  VI  VI  IV (VII)  IV (VII) | 45  16  2  2  34  34  34  34  2  34  2  2  2  18  2  18  2  2  2  2  34  49  32  57  32  13  13  13  50  2  61  34  44  34  34  34  2  34  2  2  71  30  30  30  30  30  30  30  30  30  30  30  30  30  30  30  30  30  56  30  62  30  17  36  36  36  36  36  17  36  17  9  58  43  8  65  41  19  5  6  63  70  58  1  60  60  23  1  1  1  20  1  60  54  2  44  25  2  35  3  37  60  60  59  17  64  66  43  4  43  7  21  46  22  30  56  30  30  48  47  19  40  58  3  31  35  13  14  39  4  58  36  9  33  53  17  45  2  2  18  18  24  55  68  18  44  51  12  28  29  69  67  42  27  15  10  52  68  11  26 | 1  9  1  1  1  1  1  1  1  1  1  1  1  1  1  1  1  1  1  1  1  14  14  14  14  12  12  14  3  17  17  17  17  17  17  17  17  6  17  17  9  9  9  9  9  9  9  9  9  9  9  9  9  9  9  9  9  9  9  9  18  9  36  36  36  36  36  36  36  36  36  36  10  12  22  5  9  9  9  9  10  18  18  32  37  37  13  37  37  32  24  34  29  1  17  17  11  17  17  4  7  2  2  36  36  36  36  3  16  11  28  28  28  26  9  9  9  9  9  9  9  8  23  4  11  17  14  3  36  3  2  36  31  5  15  36  1  1  1  1  1  9  9  9  1  1  25  32  33  30  21  1  20  38  19  16  27  9  20  38 | 20  20  2  2  20  20  20  2  2  20  20  20  2  2  2  2  2  2  2  2  2  2  2  2  2  2  2  2  1  29  2  2  14  14  2  2  2  2  2  2  1  1  1  1  1  1  1  1  1  1  1  1  1  1  1  1  1  13  13  13  1  4  1  12  1  1  1  1  1  1  19  13  12  1  6  15  13  13  16  25  14  27  2  3  3  3  22  3  22  22  17  13  3  2  2  2  4  14  14  1  1  7  16  12  1  12  30  1  38  12  4  4  24  4  12  1  33  12  15  15  1  15  3  3  15  17  14  12  27  35  34  12  23  14  37  1  2  20  2  2  2  25  28  13  2  2  32  10  21  8  11  9  39  26  13  18  36  31 | 3  9  9  9  9  9  9  9  9  9  9  9  9  22  9  9  9  9  9  9  9  9  9  9  9  9  9  9  10  3  9  9  9  9  9  9  9  9  9  9  10  10  10  10  10  10  10  10  10  10  10  10  10  10  10  10  10  10  10  10  10  10  1  10  10  10  10  10  1  10  3  19  23  10  2  2  2  6  1  1  9  4  9  9  9  9  4  9  1  1  10  1  9  9  9  9  1  9  9  17  10  1  1  1  1  1  3  1  11  1  1  1  10  1  1  1  10  1  1  1  1  1  9  17  2  11  12  1  1  1  2  1  9  9  14  14  3  3  9  9  9  1  23  10  9  9  5  13  21  21  8  18  20  15  20  7  16  16 | 2  2  1  30  1  1  16  16  1  30  30  30  1  1  1  1  1  1  1  1  1  1  29  29  1  1  9  1  10  1  1  1  1  1  1  1  1  1  1  1  29  8  29  29  29  8  29  29  5  5  29  5  5  20  5  5  5  29  8  29  3  1  17  4  4  4  4  4  17  28  17  28  18  18  3  13  17  17  2  27  6  22  16  29  5  28  18  28  12  12  7  3  2  25  1  16  17  23  3  28  33  3  34  17  17  2  17  11  16  2  28  16  1  20  29  5  20  21  1  17  6  2  2  36  15  5  19  4  19  2  19  37  26  24  17  2  1  16  1  16  14  7  4  1  1  35  40  31  31  38  38  44  42  41  41  39  32  43  43 | 14  1  1  1  1  1  25  1  1  14  14  1  1  1  1  14  39  1  1  1  1  34  1  1  26  22  26  35  23  10  26  10  1  26  10  10  1  1  1  1  4  8  4  25  4  4  38  4  4  4  4  4  33  32  4  4  4  4  36  4  27  14  15  15  15  15  15  15  19  1  15  14  18  6  6  14  1  15  1  1  18  1  1  1  1  1  1  1  1  1  7  1  2  1  1  1  13  13  21  13  16  16  16  15  15  15  15  29  2  2  4  4  2  4  4  13  4  13  2  17  13  2  2  13  13  9  20  2  17  2  37  14  2  32  17  1  1  1  1  11  4  2  2  14  3  31  31  30  30  3  3  12  24  40  40  15  15  12  12 | 93  33  6  6  69  69  71  68  6  70  10  9  6  41  6  40  7  6  6  6  68  97  65  106  66  27  28  29  98  13  116  73  91  74  73  73  11  72  11  11  127  55  54  56  54  54  59  54  54  54  54  54  58  57  54  54  54  62  105  62  117  60  34  80  78  78  78  78  35  77  37  23  108  88  21  120  84  43  18  19  118  126  109  1  115  115  47  4  5  2  44  3  114  102  11  90  49  12  75  14  81  112  113  111  34  119  121  86  17  87  20  45  94  46  61  104  63  61  96  95  42  83  110  15  64  76  30  31  82  16  107  79  22  67  101  36  92  8  6  38  39  48  103  123  40  89  99  26  52  53  125  122  85  51  32  24  100  124  25  50 |

***** This strain was designated as *Salmonella* Typhi. After PCR-RFLP and Salmonella standard molecular serotyping we confirmed its serotype as S. Typhimurium.
