# Supplementary material for: Differentiation of Salmonella strains from the SARA, SARB and SARC reference collections by using three genes PCR-RFLP and the 2100 Agilent Bioanalyzer
Source: Front Microbiol. 2014 Aug 11;5:417. doi: 10.3389/fmicb.2014.00417 (PMC4127528; doi:10.3389/fmicb.2014.00417)
Supplement: Supplementary file 6 [file DataSheet6.DOCX]

Supplementary Table 6- Comparison of typeability by the different methods and analyses

| **Serotype**  **or**  **Subspecies** | Total | PCR-RFLP | | | | Concatenated 7  housekeeping genes  (MLST) | | | | Concatenated  *fliC*, *gnd* and *mutS*  genes sequences | | | | MLST  Sequence  Type  (ST) | PCR-RFLP  Restriction Type  (RT) |
| --- | --- | --- | --- | --- | --- | --- | --- | --- | --- | --- | --- | --- | --- | --- | --- |
|  |  | Clustered | Single | Diff. | Not Diff. | Clustered | Single | Diff. | Not  Diff. | Clustered | Single | Diff. | Not  Diff. | Num. STs | Num. RTs |
| **Agona**  **Anatum**  ***arizonae***  ***bongori***  **Brandernburg**  **Choleraesuis**  **Decatur**  **Derby**  ***diarizonae***  **Dublin**  **Dublin/Enteritidis**  **Duisburg**  **Emek**  **Enteritidis**  **Gallinarum**  **Haifa**  **Heidelberg**  ***houtenae***  **Indiana**  ***indica***  **Infantis**  **Javiana**  **Limete**  **Manhattan**  **Miami**  **Montevideo**  **Muenchen**  **Newport**  **Oranienburg**  **Panama**  **Paratyphi A**  **Paratyphi B**  **Paratyphi C**  **Reading**  **Rubislaw**  **Saintpaul**  ***salamae***  **Schwarzengrund**  **Sendai**  **Senftenberg**  **Stanley**  **Stanleyville**  **Thompson**  **Typhi**  **Typhimurium**  **Typhisuis**  **Wien** | 2  1  2  2  1  2  4  3  2  2  1  1  1  4  3  1  13  4  1  2  2  2  2  3  2  2  11  3  1  1  1  24  2  1  1  10  2  1  1  1  1  1  1  2  27  1  2 | 2  1  2  2  1  2  4  3  2  2  1  1  0  4  3  1  13  4  1  2  2  2  2  1  1  2  11  3  0  1  1  24  2  1  1  10  2  1  1  1  1  0  0  0  26  0  2 | 0  0  0  0  0  0  0  0  0  0  0  0  1  0  0  0  0  0  0  0  0  0  0  2  1  0  0  0  1  0  0  0  0  0  0  0  0  0  0  0  0  1  1  2  1  1  0 | 2  0  2  2  0  1  3  3  2  0  0  0  1  3  0  0  12  4  0  2  2  0  0  2  1  2  9  0  1  0  0  23  0  0  0  7  2  0  0  0  0  1  1  2  25  1  2 | 0  1  0  0  1  1  1  0  0  2  1  1  0  1  3  1  1  0  1  0  0  2  2  1  1  0  2  3  0  1  1  1  2  1  1  3  0  1  1  1  1  0  0  0  2  0  0 | 2  1  2  2  1  2  3  3  2  2  0  0  1  2  3  1  13  3  1  1  0  2  2  3  1  2  11  2  0  1  1  24  2  1  0  8  2  0  1  1  0  1  0  2  25  0  1 | 0  0  0  0  0  0  1  0  0  0  1  1  0  2  0  0  0  1  0  1  2  0  0  0  1  0  0  1  1  0  0  0  0  0  1  2  0  1  0  0  1  0  1  0  2  1  1 | 0  0  2  2  0  2  3  0  2  2  1  1  0  2  0  0  5  3  0  1  2  2  2  2  1  2  9  3  1  0  0  22  2  0  1  7  2  1  0  0  1  0  1  2  26  1  1 | 2  1  0  0  1  0  1  3  0  0  0  0  1  2  3  1  8  1  1  1  0  0  0  1  1  0  2  0  0  1  1  2  0  1  0  3  0  0  1  1  0  1  0  0  1  0  1 | 1  1  2  2  1  2  4  2  2  0  1  1  1  4  3  1  10  4  1  0  2  2  1  1  1  2  8  2  1  1  1  18  2  0  0  10  1  0  0  0  1  0  1  2  23  1  2 | 1  0  0  0  0  0  0  1  0  2  0  0  0  0  0  0  3  0  0  2  0  0  1  2  1  0  3  1  0  0  0  6  0  1  1  0  1  1  1  1  0  1  0  0  4  0  0 | 1  0  2  2  0  2  0  1  2  2  0  0  0  0  0  0  5  4  0  2  0  2  1  2  1  0  9  3  0  0  0  20  0  1  1  6  1  1  1  1  0  1  0  2  12  0  0 | 1  1  0  0  1  0  4  2  0  0  1  1  1  4  3  1  8  0  1  0  2  0  1  1  1  2  2  0  1  1  1  4  2  0  0  4  1  0  0  0  1  0  1  0  15  1  2 | 1  1  0*  0*  1  2  3  3  2  2  1  1  1  3  2  1  2  1*  1  1*  2  1  1  2  2  2  5  3  1  1  1  7  2  1  1  4  0*  1  1  1  1  1  1  2  4  1  2 | 2  1  2  2  1  2  4  3  2  2  1  1  1  4  3  1  8  4  1  2  2  2  2  3  2  2  7  3  1  1  1  12  2  1  1  10  2  1  1  1  1  1  1  2  16  1  2 |
| **Total**  **(%)** | 160 | 149 (93.1%) | 11  (6.9%) | 118  (73.8%) | 42  (26.2%) | 137  (85.6%) | 23  (14.4%) | 117  (73.1%) | 43  (26.9%) | 126  (78.8%) | 34  (21.2%) | 88  (55.0%) | 72  (45.0%) | 150  (93.8%) | 160  (100%) |

* No STs were obtained for some of the strains: *S*. *arizonae* (2/2), *S*. *bongori* (2/2), *S*. *houtenae* (3/4), *S*. *indica* (1/2), and *S*. *salamae* (2/2)

Diff.= differentiation; No Diff. = No differentiation
