# Supplementary material for: Differentiation of Salmonella strains from the SARA, SARB and SARC reference collections by using three genes PCR-RFLP and the 2100 Agilent Bioanalyzer
Source: Front Microbiol. 2014 Aug 11;5:417. doi: 10.3389/fmicb.2014.00417 (PMC4127528; doi:10.3389/fmicb.2014.00417)
Supplement: Supplementary file 7 [file DataSheet7.DOC]

**Supplementary Table 7-Epidemiological Concordance**

| Serovar /subgroup  (Total) | Total  strains | | Source distribution | | | ST | | RT | |
| --- | --- | --- | --- | --- | --- | --- | --- | --- | --- |
|  | Source | Unknown | Origin | Num. | % | Total | Unique | Total | Unique |
| Typhimurium (27) | 16 | 11 | Human  Animal  Unknown | 6  10  11 | 22.0  37.0  41.0 | 1  3  2 | 0  2  1 | 5  6  9 | 4  3  6 |
| Paratyphi B (24) | 24 | 0 | Human  Animal  Food  Environmental | 15  2  3  4 | 62.5  8.3  12.5  16.7 | 5  2  3  2 | 2  0  1  1 | 10  2  3  3 | 7  0  1  1 |
| Heidelberg (13) | 8 | 5 | Human  Animal  Unknown | 2  6  5 | 15.4  46.1  38.5 | 2  1  2 | 0  0  0 | 2  5  4 | 1  3  2 |
| Muenchen (11) | 10 | 1 | Human  Animal  Unknown | 8  2  1 | 72.7  18.2  9.1 | 4  1  1 | 4  0  0 | 5  2  1 | 4  1  1 |
| Saintpaul (10) | 7 | 3 | Human  Unknown | 7  3 | 70.0  30.0 | 4  3 | 2  1 | 7  3 | 7  3 |
| Decatur (4) | 1 | 3 | Animal  Unknown | 1  3 | 25.0  75.0 | 1  3 | 0  2 | 1  3 | 1  3 |
| Enteritidis (4) | 1 | 3 | Human  Unknown | 1  3 | 25.0  75.0 | 1  2 | 1  2 | 1  3 | 1  3 |
| *houtenae* (4) | 3 | 1 | Human  Animal  Unknown | 2  1  1 | 50.0  25.0  25.5 | 1*  -*  -* | -*  -*  -* | 2  1  1 | 2  1  1 |
| Derby (3) | 3 | 0 | Animal | 3 | 100.0 | 3 | 3 | 3 | 3 |
| Gallinarum (3) | 1 | 2 | Human  Unknown | 1  1 | 50.0  50.0 | 1  1 | 1  1 | 1  2 | 1  2 |
| Manhattan (3) | 3 | 0 | Human | 3 | 100.0 | 2 | 2 | 3 | 3 |
| Newport (3) | 3 | 0 | Human  Animal | 2  1 | 66.7  33.3 | 2  1 | 2  1 | 2  1 | 2  1 |
| Agona (2) | 1 | 1 | Environmental  Unknown | 1  1 | 50.0  50.0 | 1  1 | 0  0 | 1  1 | 1  1 |
| *arizonae* (2) | 2 | 0 | Human  Animal | 1  1 | 50.0  50.0 | -*  -* | -*  -* | 1  1 | 1  1 |
| *bongori* (2) | 2 | 0 | Animal | 2 | 100.0 | -* | -* | 2 | 2 |
| Choleraesuis (2) | 1 | 1 | Animal  Unknown | 1  1 | 50.0  50.0 | 1  1 | 1  1 | 1  1 | 1  1 |
| *diarizonae* (2) | 2 | 0 | Human | 2 | 100.0 | 2 | 2 | 2 | 2 |
| Dublin (2) | 2 | 0 | Animal | 2 | 100.0 | 2 | 2 | 2 | 2 |
| *indica* (2) | 0 | 2 | Unknown | 2 | 100.0 | 1* | -* | 2 | 2 |
| Infantis (2) | 1 | 1 | Human  Unknown | 1  1 | 50.0  50.0 | 1  1 | 1  1 | 1  1 | 1  1 |
| Javiana (2) | 2 | 0 | Human | 2 | 100.0 | 1 | 1 | 2 | 2 |
| Limete (2) | 2 | 0 | Human | 2 | 100.0 | 1 | 1 | 2 | 2 |
| Miami (2) | 2 | 0 | Human | 2 | 100.0 | 2 | 2 | 2 | 2 |
| Montevideo (2) | 2 | 0 | Human | 2 | 100.0 | 2 | 2 | 2 | 2 |
| Paratyphi C (2) | 1 | 1 | Human  Unknown | 1  1 | 50.0  50.0 | 1  1 | 1  1 | 1  1 | 1  1 |
| *salamae* (2) | 1 | 1 | Human  Unknown | 1  1 | 50.0  50.0 | -*  -* | -*  -* | 1  1 | 1  1 |
| Typhi (2) | 2 | 0 | Human | 2 | 100.0 | 2 | 2 | 2 | 2 |
| Wien (2) | 0 | 2 | Unknown | 2 | 100.0 | 2 | 2 | 2 | 2 |

* No ST reported in the MLST Database
