# Supplementary material for: Differentiation of Salmonella strains from the SARA, SARB and SARC reference collections by using three genes PCR-RFLP and the 2100 Agilent Bioanalyzer
Source: Front Microbiol. 2014 Aug 11;5:417. doi: 10.3389/fmicb.2014.00417 (PMC4127528; doi:10.3389/fmicb.2014.00417)
Supplement: Supplementary file 8 [file Image1.PDF]

gnd-F1 primer 3’- G G T C G A T T G A A T T A A G T C C G C G T C-5’

C13	*S. indica* (VI)	1261	5’-CGCGCCGCAGTACTG	C C A G C T A A C T T A A T T C A G G C G C A G	CGTGACTACTT-3’	1310
C1	*S.* Typhimurium (I)		CGCGCCGCAGTACTG	C C A G C T A A C T T A A T T C A G G C G C A G	CGTGACTACTT	
C2	*S.* Typhi (I)		CGCGCCGCAGTACTG	C C A G C T A A C T T A A T T C A G G C G C A G	CGTGACTACTT	
C8	*S. diarizonae* (IIIb)		CGCGCCGCAGTACTG	C C A G C T A A C C T G A T T C A G G C G C A G	CGTGACTACTT	
C7	*S. diarizonae* (IIIb)		CGCGCCGCAGTACTG	C C A G C T A A C C T G A T T C A G G C G C A G	CGTGACTACTT	
C14	*S. indica* (VI)		CGCGCCGCAGTACTG	C C A G C T A A T C T G A T T C A G G C G C A G	CGTGACTACTT	
C16	*S. houtenae* (VII)		CGCGCCGCGGTACTG	C C A G C T A A C C T G A T T C A G G C G C A G	CGTGACTACTT	
C15	*S. houtenae* (VII)		CGCGCCGCGGTACTG	C C A G C T A A C C T G A T T C A G G C G C A G	CGTGACTACTT	
C10	*S. houtenae* (IV)		CGCGCCGCGGTACTG	C C A G C T A A C C T G A T T C A G G C G C A G	CGTGACTACTT	
C9	*S. houtenae* (IV)		CGCGCCGCGGTACTG	C C A G C T A A C C T G A T T C A G G C C C A G	CGTGACTACTT	
C4	*S. salamae* (II)		CGCGCCGCAGTACTG	C C A G C T A A C C T G A T T C A G G C G C A G	CGTGACTATTT	
C3	*S. salamae* (II)		CGCGCCGCAGTACTG	C C A G C T A A C C T G A T T C A G G C G C A G	CGTGACTATTT	
C6	*S. arizonae* (IIIa)		CGTGCCGCGGTACTG	C C A G C T A A C C T G A T T C A G G C G C A G	CGTGACTATTT	
C5	*S. arizonae* (IIIa)		CGTGCCGCGGTACTG	C C A G C T A A C C T G A T T C A G G C G C A G	CGTGACTATTT	
C12	*S. bongori* (V)		CGCGCTGCGATACTG	C C G G C T A A C C T G A T T C A G G C T C A G	CGCGACTATTT	
C11	*S. bongori* (V)		CGCGCTGCGATACTG	C C G G C T A A C C T G A T T C A G G C T C A G	CGCGACTATTT	

Figure S1. Annealing of gnd F-1 primer with gnd gene sequences from the SAR C Reference Collection
